# NuMA1 controls myonuclear motility in striated skeletal muscle through AMPK activity and is impaired in Duchenne muscular dystrophy

**DOI:** 10.1038/s41419-026-08987-5

**Published:** 2026-06-17

**Authors:** Nathalie Couturier, Léa Castellano, Alireza Ghasemizadeh, Emilie Christin, Muriel Sébastien, Caroline E. Brun, Damien Caillol, Céline Malleval, Alexandre Janin, Gaëtan Juban, Alexis Osseni, Jean-Luc Thomas, Stéphane Koenig, Jessica Brunetti, Alexandra Kraut, Yohann Couté, Nathalie Streichenberger, Rémi Mounier, Vincent Gache

**Affiliations:** 1 Institut NeuroMyoGène, Physiopathologie et Génétique du Neurone et du Muscle, UMR CNRS 5261 – INSERM U1315, Université Claude Bernard Lyon 1, Lyon, France; 2 Department of Cell Physiology and Metabolism, Geneva Medical Center, Geneva, Switzerland; 3 CEA, INSERM, UA13 BGE, CNRS, CEA, UAR2048, Grenoble Alpes University, Grenoble, France; 4 Department of Neuropathology, Groupe Hospitalier Est, Hospices Civils de Lyon, Bron, France

**Keywords:** Microtubules, Mechanisms of disease, Diagnostic markers, Differentiation, Disease model

## Abstract

Skeletal muscle consists of a bundle of thousands of post-mitotic multinucleated cells (i.e., myofibers), in which myonuclei are evenly spaced and positioned at the periphery. This myonuclear positioning shapes myonuclear domain (MND) in myofibers, is essential for the transcriptional integrity of myofibers, is driven by the cytoskeleton and associated proteins and is required for proper myofiber functions. In numerous muscle diseases (i.e., myopathies), alteration of myonuclei localization contributes to myofiber dysfunction, supporting the need to better understand the fundamental mechanisms that regulate myonuclei dynamics in differentiated myofibers. In this study, we show that in Duchenne muscular dystrophy (DMD) myofibers, myonuclei are more dynamic and contribute to the failure in MND settings, suggesting enhanced myonuclear motility impacts myonuclear distribution. To identify new actors in MND settings, we performed a mass spectrometry (MS)-based proteomic analysis to identify microtubule-associated proteins (MAPs) in myotubes/myofibers and performed a siRNA screening on candidates. This approach highlighted NuMA1 as a new factor controlling myoblast fusion and myonuclear positioning through the control of nuclear-microtubule-organizing-center (n-MTOC) integrity and microtubule network orientation. Strikingly, while NuMA1 is restrained to myonuclei in mononucleated myoblasts, it progressively accumulates in the cytoplasm during muscle cell differentiation, preferentially with microtubule (MT) nucleation spots at the vicinity of the nuclear membrane. We identified that AMP Kinase activity has an essential role in NuMA1 nuclear/cytoplasmic accumulation through the specific phosphorylation on serine-1853 and the ability of myonuclei to accumulate NuMA1 is correlated to their motility in myofibers. Finally, we show that nuclear NuMA1 content is increased in DMD patients and *mdx* mouse model, contributing to more dynamic myonuclei that can be manipulated pharmacologically through the control of AMPK activity. Altogether, our data identifies a novel mechanism by which nuclear sequestration of a MAP allows to couple nuclear positioning and motion to MT organization along skeletal muscle differentiation.

## Introduction

The positioning of nuclei in large post-mitotic syncytium, such as in skeletal muscle cells, is a challenging process as these cells contain thousands of nuclei in which proper positioning controls muscle cells functionality [1]. Myonuclear mispositioning is a hallmark of several muscle diseases, including centronuclear myopathies (CNM) and Duchenne muscular dystrophy (DMD), and has been associated with myofiber dysfunction [2–5]. Indeed, nuclei mispositioning is associated with transcriptional and translational homeostasis disruption, as nuclei support local protein synthesis, required for sarcomere maintenance and repair [6–9]. In addition, nuclei positioning can affect cytoskeletal organization through their mechanical association with the microtubule and actin networks [10–12]. Defective nuclear–cytoskeletal coupling compromises force transmission and alters the mechanical properties of muscle fiber [13, 14]. Finally, proper nuclear distribution is particularly critical during myofiber maturation and regeneration, where spatially coordinated gene expression is required to support growth and functional recovery [3, 15, 16]. Myonuclear positioning is a key determinant of muscle function and mechanisms that ensure correct nuclear organization need to be characterized to better appreciate their physiological relevance.

Nuclear positioning is an active process, controlling cells fate determination and involving mechanical interplays between nuclear envelope proteins and cytoskeleton networks [17]. Microtubule (MT) network appears as a key player in the regulation of nuclear positioning settings in the majority of cells [18]. Most differentiated cells, including muscle cells, epithelial cells, and neurons, set up “non-centrosomal” MTOC (ncMTOC), allowing the nucleation/assembly of MTs at a certain distance from the nucleus, leading to alternative patterns, orientation/polarization of the MT network [19–21]. The expression of various MT-interacting proteins along skeletal muscle cells differentiation carries out myonuclei positioning through the control/maintenance of newly formed MT network architecture [22]. Previously, a limited number of MAPs (Microtubule Associated Proteins) and molecular motors have been identified as controlling myonuclei motion and thus myonuclei spreading in developing muscle cells [1, 23–26].

We showed that in mature myofibers, enhanced myonuclei velocity is correlated with a delay in the formation/maturation of organelles such as the sarcoplasmic reticulum and can ultimately lead to internalized myonuclei within myofibers, associated with transcriptional activity misregulation [3, 4]. In addition, myonuclei displacement into mature myofibers can reveal local ongoing processes such as muscle cell reparation [9]. However, it is still puzzling how myonuclei remain anchored in specific localizations along mature mammalian skeletal muscle fibers and what are the functional consequences of positioning/shape alterations. In pathological conditions such as in DMD, it is common to find merging/aggregated myonuclei with various shapes and orientations along mature myofiber [27–29]. Interestingly, myonuclei velocity or time-in-motion in developing myofibers fluctuates depending on the developmental phase, the presence of myofibrils and are far from being homogenous between myonuclei in the same myotubes/myofibers and so far, no specific marker has been identified that reveals myonuclei motility [1, 3, 23, 30]. Here, we confirm the correlation between alteration of myonuclei velocity and muscle pathological conditions (DMD) and provide a new mechanism for the regulation of nuclear movement inside myofibers and the identification of marker reflecting myonuclei motility.

## Results

### Myonuclear velocity/displacement is enhanced in Duchenne muscular dystrophy myofibers

**Fig. 1:**
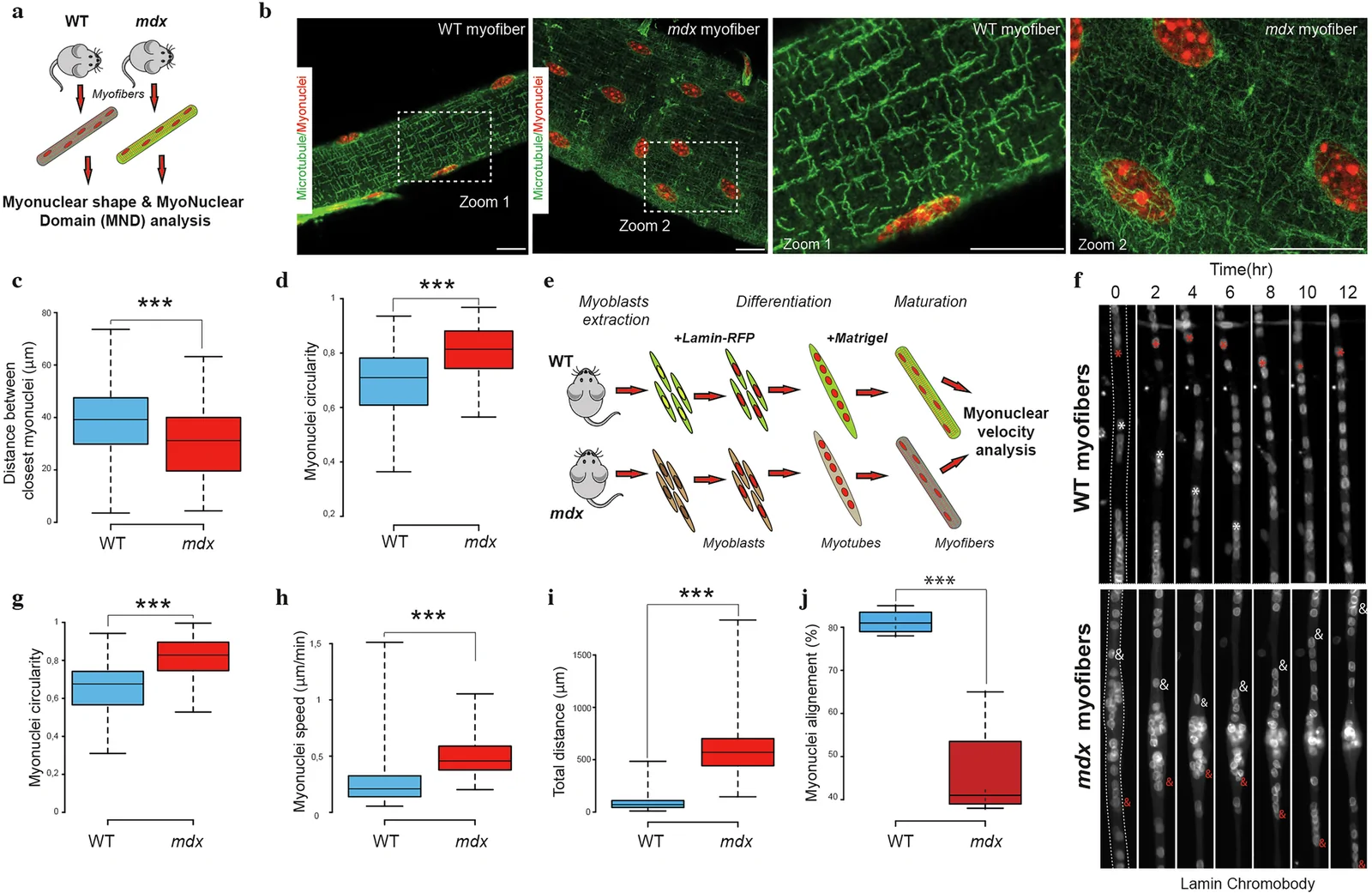
Myonuclear dynamics and shape in Duchenne muscular dystrophy myofibers. **a** Scheme representation of the experimental procedure for isolating *Tibialis Anterior* myofibers. **b** Representative immunofluorescence images of isolated *Tibialis Anterior* myofibers from 2-month-old WT or *mdx* mice, stained for MTs (green) and myonuclei (red) (Scale bar: 10 µm). **c, d** Quantification of the distance between nearest myonuclei (µm) (*n* = 3 independent WT and *mdx* mice, 208 WT and 250 *mdx* measurements) (**c**) and myonuclei circularity (*n* = 3 independent WT and *mdx* mice, 255 WT and 295 *mdx* myonuclei quantified) (**d**). **e** Schematic of the in vitro experimental procedure for forming WT or *mdx* primary myotubes after Lamin-RFP-Chromobody^®^ transfection to track myonuclear movements. **f** Kymograph from 24 h timelapse of 5-day WT or *mdx* primary myofibers transfected with Lamin-RFP-Chromobody^®^. First frame shows a myofiber; images acquired every 15 min. **g** Quantification of myonuclear circularity in 3-day WT or *mdx* primary myotubes in vitro (*n* = 3 independent primary cultures, 126 WT and 157 *mdx* myonuclei analyzed). **h, i** Quantification of myonuclear speed (µm/min) (**h**), and total displacement (µm) (**i**) in 5-day WT or *mdx* primary myofibers in vitro (*n* = 3 independent primary cultures, 300 WT and 239 *mdx* myonuclei tracked). **j** Quantification of the percentage of myotubes with aligned myonuclei in 3-day WT or *mdx* primary myotubes in vitro (*n* = 4 independent primary cultures). Graphical boxes showed the total data population represented by the higher and the lower point of the population. The median is represented. Unpaired T-test was performed between groups. Each *p*-value is mentioned in the figure legend of each graph, ns (not significant) *p < 0.05, **p < 0.01, and ***p < 0.001.

DMD is caused by mutations in the *DMD* gene, encoding the dystrophin protein, previously identified as a MAP [31]. Indeed, in *mdx* mice, a well-established model of human DMD, MT network organization in skeletal muscle cells is dramatically altered [32]. To investigate the relationship between nuclear positioning and MT network impairment, we isolated *Tibialis Anterior* muscle fibers from 2-month-old WT and *mdx* mice (Fig. 1a–d). We first confirmed MT network disorganization in *mdx* muscle fibers (Fig. 1b) and found that the distance between myonuclei in *mdx* muscle fibers was decreased by more than 20%, reflecting a reduction in MND size (Fig. 1c). Interestingly, myonuclei circularity is increased by more than 15% in *mdx* muscle fibers compared with the WT condition, indicating altered global mechanical tension applied on each myonucleus, which consequently appear rounder (Fig. 1b, d).

To determine whether myonuclei shape alterations reflect a failure in myonuclear velocity that impairs MND settings [3], we isolated primary myoblasts from WT and *mdx* mice pups (age P4-6) and transfected the cells with a TagRFP-lamin-chromobody® plasmid to visualize myonuclei shape and displacement. Myoblasts were further induced to fuse to form myotubes, and were maturated into myofibers using a Matrigel® sandwich culture [33] (Fig. 1e). We next performed time-lapse confocal microscopy (Fig. 1f) and tracked myonuclei in these in vitro myofibers (Fig. 1g–j). First, quantification of myonuclear circularity revealed that deformation of myonuclei is an early event, with a nearly 10% increase in myonuclei circularity in *mdx* myofibers compared to controls (Fig. 1g). Myonuclear displacement analysis showed that, in control condition, myonuclei alternate between phases of movement and pauses. Myonuclear motion occurred at a median speed of 0.21 ± 0.01 µm/min, with pauses lasting approximately 5 h per day, resulting in a median daily displacement of around 150 µm (Fig. 1h, i and Supplementary Fig. 1a, b). In *mdx* myofibers, pause duration was decreased by more than half (Supplementary Fig. 1b), while median speed increased to 0.43 ± 0.01 µm/min, yielding an average daily displacement of up to 500 µm (Fig. 1h, i and Supplementary Fig. 1a, b). Interestingly, this enhancement of myonuclei velocity correlated with a failure to correctly align myonuclei, which appeared aggregated in more than half of myofibers (Fig. 1j). Altogether, our data suggests that rounder myonuclei reflect more displacement of myonuclei in mature myofibers and confirms that the increased myonuclear velocity observed in *mdx* myofibers leads to alterations in MND organization in myofibers.

### NuMA1 is required for myonuclear positioning during myofiber formation

Myonuclei displacement in myofibers has primarily been linked to interactors of the MT network [1, 3, 9, 23, 34]. To identify new factors that interact early with, and regulate, MT network architecture while contributing to the MND organization during myofiber formation, we purified MAPs from either 3-day primary myotubes or 10-day primary myofibers. MT-interacting proteins were identified using MS-based proteomics, enabling classification according to their enrichment in immature (myotubes) or mature (myofibers) stages (Supplementary Fig. 1c–e and Supplementary Table 1). Among the 2037 proteins identified, we selected 66 candidates and tested their involvement in myonuclear spreading using pools of 3 specific siRNAs (Supplementary Table 2). Approximately half of the candidates, when silenced, caused defective myonuclear alignment during the early steps of myotube formation (Supplementary Fig. 1e). Notably, this targeted siRNA screen revealed a critical role for *Numa1*, whose down-regulation produced a more severe defect than the absence of Kif5b, a previously described molecular motor regulating myonuclear spreading in myotubes and myofibers [1, 23, 35, 36].

**Fig. 2:**
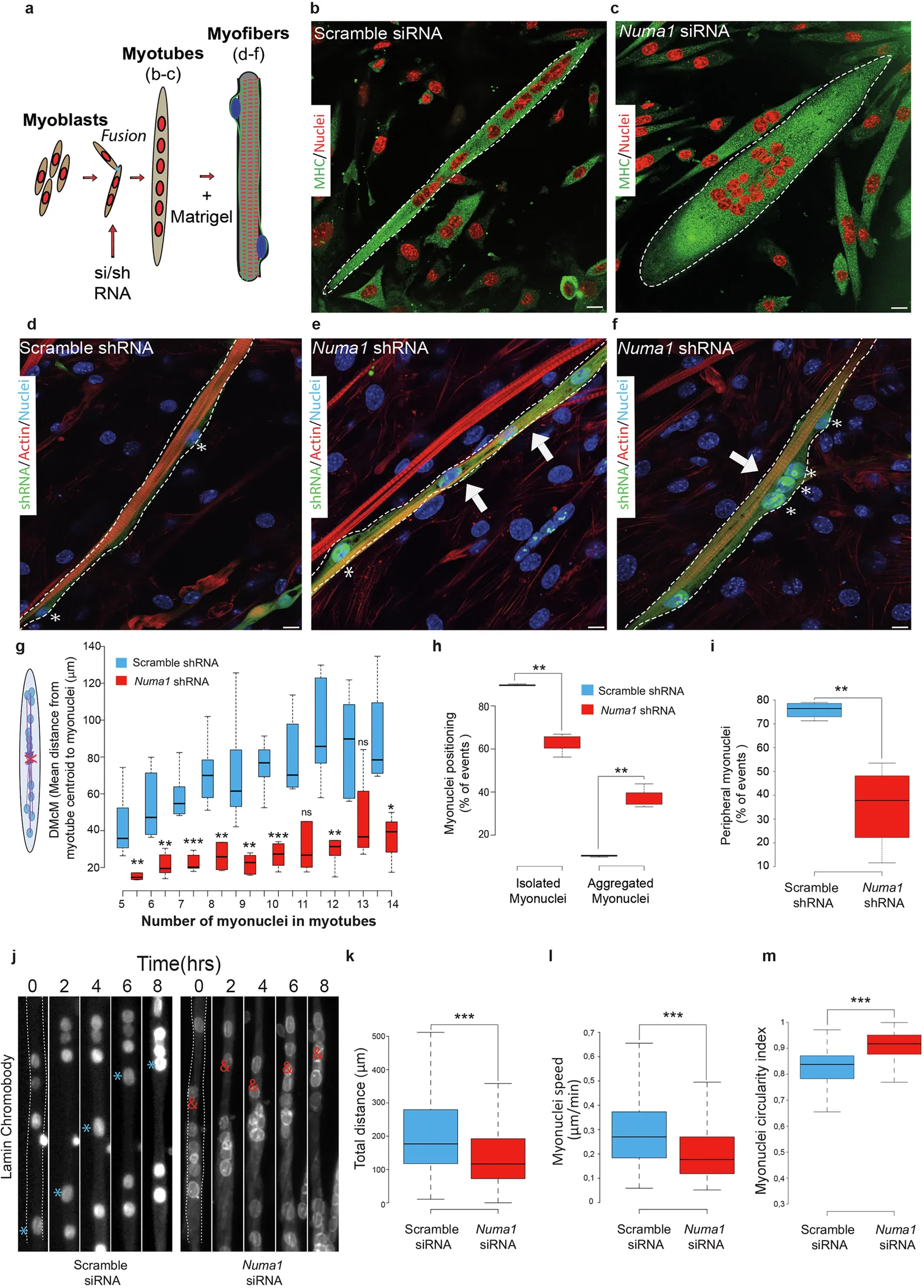
*Numa1* down-regulation affects myonuclei positioning and dynamics during myofiber formation in vitro. **a** Schematic of in vitro experimental procedure of 3-day primary myotubes and 10-day primary myofibers after siRNA or shRNA transfection. **b, c** Representative immunofluorescence images of 3-day primary myotubes transfected with scramble (**b**) or *Numa1* (**c**) siRNA, stained for Myosin Heavy Chain (MHC, green) and myonuclei (red) (Scale bar: 10 µm). **d**–**f** Representative immunofluorescence images of 10-day primary myofibers after scramble (**d**) or *Numa1* shRNA (**e**, **f**) transfection, stained for shRNA plasmid expression (green), actin (red), and nuclei (blue). Arrows indicate non-peripheral myonuclei; asterisks indicate peripheral myonuclei (Scale bar: 10 µm). **g** Quantification of mean Distance between Myotubes centroid and Myonuclei (DMcM) in 3-day primary myotubes after scramble or *Numa1* siRNA transfection (*n* = 6 independent primary cultures, 618 scramble shRNA and 687 *Numa1* shRNA distances measured). **h, i** Quantification of isolated and aggregated myonuclei (*n* = 3 independent primary cultures, 1452 analyzed events in scramble and 2009 in Numa1 knockdown condition) (**h**) and peripheral myonuclei frequency (*n* = 4 independent primary cultures, 966 quantified events in scramble and 760 in Numa1 knockdown conditions) (**i**) in 10-day primary myofibers after scramble or *Numa1* shRNA transfection in vitro. **j** Representative kymograph from timelapse imaging of 5-day primary myofibers co-transfection with Lamin-chromobody®-RFP and scramble or *Numa1* siRNA. The first frame of each representative 16 h movie shows a myofiber. Myonuclei were tracked for 16 h with one image acquired every 20 min. **k, l** Quantification of the total distance traveled by myonuclei (µm) (**k**), and myonuclei speed (µm/min) (**l**) in 5-day primary myofibers after scramble or *Numa1* siRNA transfection in vitro (*n* = 2 independent primary cultures, 214 myonuclei tracked in scramble and 173 in *Numa1* siRNA). **m** Quantification of myonuclei circularity *in* 3-day primary myotubes after scramble or *Numa1* siRNA transfection in vitro (*n* = 3 independent primary cultures, 126 myonuclei in scramble condition and 137 in *Numa1* siRNA). Graphical boxes showed the total data population represented by the higher and the lower point of the population. The median is represented. Unpaired T-test was performed between groups. Each *p*-value is mentioned in the figure legend of each graph, ns (not significant) *p < 0.05, **p < 0.01 and ***p < 0.001.

NuMA1 is well known as a key player in cell division, particularly in mitotic spindle orientation [37, 38], but no specific role has been described in post-mitotic muscle cells. We next confirmed that efficient si/shRNA-mediated depletion of *Numa1* in either primary mouse myoblasts or immortalized murine myoblasts inhibited myonuclear spreading in both myotubes and myofibers (Fig. 2a–i and Supplementary Fig. 2a–e). In control condition, quantification of the “mean Distance between Myotubes centroid and Myonuclei” (DMcM) progressively increased as new myonuclei were incorporated into myotubes (Fig. 2g), reflecting physiological myonuclei spreading during myotube growth and elongation (Supplementary Fig. 2c). In contrast, *Numa1* down-regulation prevented DMcM from increasing, leaving it relatively stables despite the incorporation of new myonuclei. This confirmed a precocious myonuclear aggregation phenotype, preferentially at the myotube center (Fig. 2g), and was associated with impaired elongation (Supplementary Fig. 2c). We confirmed the specific involvement of NuMA1 in this myonuclei spreading process by rescue experiments using an siRNA-resistant human-NuMA1 construct, which restored proper myonuclei alignment (Supplementary Fig. 2f, g). Long-term *Numa1* depletion in myofibers also resulted in persistent myonuclear aggregation, with 40% of myofibers displaying this phenotype compared with only 10% in controls, along with reduced peripheral positioning of myonuclei (Fig. 2d–f, h, i). Because myonuclei aggregation in myotubes can also arise from enhanced fusion [4], we assessed the number of myonuclei per myotube (fusion index) in *Numa1* depleted condition or NuMA1 overexpression condition (Supplementary Fig. 2h). Unexpectedly, low NuMA1 levels favored myoblast fusion, whereas NuMA1 over-expression slowed down fusion. These findings suggest that NuMA1 abundance also influences myoblast fusion capacity and thereby modulates the timing necessary for proper myonuclear dispersion.

### NuMA1 controls myonuclear velocity

We previously demonstrated that myonuclei aggregation correlates with the capacity of myonuclei to move [3, 4, 23]. To confirm the role of NuMA1 in regulating myonuclear velocity and exclude confounding effects from altered cell fusion, we investigated whether NuMA1 directly controls myonuclear motion. Using time-lapse confocal microscopy, we tracked myonuclei displacement in control (scramble siRNA-treated cells) and *Numa1*-depleted myofibers (Fig. 2j–l). In controls, myonuclear motion occurred at a median speed of 0.27 ± 0.01 µm/min, with pauses lasting approximately 2.5 h per day, resulting in a median daily displacement of ≈350 µm (Fig. 2k, l and Supplementary Fig. 3a). In contrast, *Numa1* depletion increased pause duration by nearly four-fold and reduced median speed to 0.18 ± 0.01 µm/min, yielding an average daily displacement of only around 100 µm (Fig. 2k, l and Supplementary Fig. 3a). Quantification of myonuclei circularity revealed a nearly 10% increase in *Numa1*-depleted myofibers compared with controls, reflected by rounder myonuclei (Fig. 2m, and Supplementary Fig. 3b, c). These data confirm that NuMA1 is involved in myonuclei displacement and suggest that the myonuclei aggregation phenotype upon *Numa1* depletion results from impaired myonuclei spreading in parallel with increased fusion events. To note, myonuclei roundness cannot be considered as a direct marker of velocity: while both *mdx* and *Numa1*-depleted myofibers displayed a 10% increase in circularity, the former was associated with enhanced myonuclei displacement (*mdx* myofibers), whereas the latter exhibited severely reduced displacement (*Numa1*-depleted myofibers) (Figs. 1 and 2). Thus, the myonuclei aggregation phenotype is closely associated with modulation of myonuclear velocity.

### NuMA1 is a nuclear protein that accumulates into the cytoplasm of myofibers and impacts myonuclear displacement

**Fig. 3:**
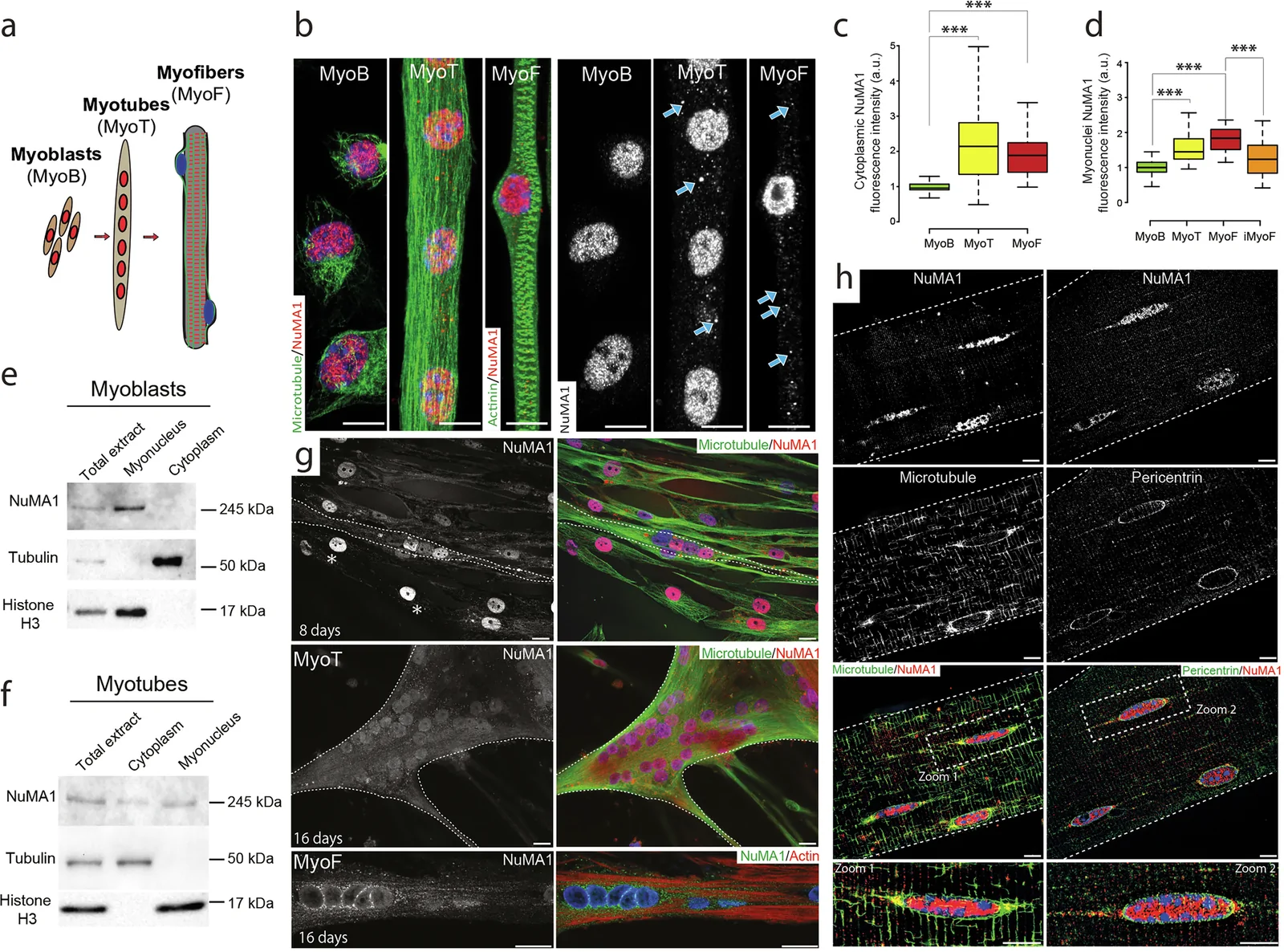
NuMA1 is a cytoplasmic protein in myofibers. **a** Schematic of the experimental procedure for in vitro formation of 3-day primary myotubes and 10-day primary myofibers. **b** Representative images of primary myoblasts (MyoB), 3-day primary myotubes (MyoT) and 10-day primary myofibers (MyoF) stained for NuMA1 (red), microtubules or actin (green) in vitro (Scale bar: 10 µm). **c, d** Quantification of cytoplasmic NuMA1 intensity (a.u.) (*n* = 3 independent primary cultures, 114 MyoB, 155 MyoT, and 114 MyoF analyzed) (**c**), and nuclear NuMA1 intensity (a.u.) (*n* = 3 independent primary cultures, 143 MyoB, 184 MyoT, and 33 MyoF and 40 iMyoF analyzed) (**d**). **e, f** Western blot after cell fractionation of primary myoblasts (**e**) and 3-day primary myotubes (**f**). Membranes probed with NuMA1, tubulin (cytoplasmic control) and histone H3 (nuclear control). **g** Representative immunofluorescence images of 8- and 16-day human primary myotubes stained for NuMA1 (red), microtubules or NuMA1 (green) and actin (red) in vitro (Scale bar: 10 µm). Asterisks mark mononucleated myoblasts. **h** High-resolution images of isolated myofibers from 2-month-old WT mice stained for NuMA1 (red), microtubules or Pericentrin (green) (Scale bar: 5 µm). Graphical boxes showed the total data population represented by the higher and the lower point of the population. The median is represented. Unpaired T-test was performed between groups. Each *p*-value is mentioned in the figure legend of each graph, ns (not significant) *p < 0.05, **p < 0.01, and ***p < 0.001.

NuMA1 has been characterized as a nuclear protein that mediates MT-membrane anchoring during mitosis [38]. This MT-related activity is enabled in mitotic cells by the absence of nuclear envelope, which allows NuMA1 to localize to the cytoplasm of mitotic cells; following nuclear envelope reformation, NuMA1 is re-imported and sequestered into the nucleus *via* its Nuclear Localization Sequence (NLS) [39]. To explore the potential mechanisms by which NuMA1 regulates myonuclear motion, we investigated its subcellular localization in muscle cells throughout myogenesis (Fig. 3a). As expected, NuMA1 was exclusively nuclear in proliferating murine primary myoblasts (Fig. 3b, MyoB) and in human primary myoblasts (Fig. 3g, asterisk). Unexpectedly immunofluorescences revealed a progressive accumulation of NuMA1 in the cytoplasm of myotubes, appearing as small puncta, concomitant with a modest increase in its overall mRNA expression during myogenesis (Fig. 3b, g, MyoT, Supplementary Fig. 3d–f). Quantification of the cytoplasmic NuMA1 signal intensity throughout myogenesis confirmed this progressive accumulation, which also occurred within the myonuclear compartment (Fig. 3c, d). In culture conditions promoting “full myofiber maturation” with well-developed sarcomeres and peripherally positioned myonuclei, remaining immature myotubes that failed to achieve this step (iMyoF) exhibited reduced NuMA1 level within myonuclei, suggesting that NuMA1 nuclear localization is dynamically regulated according to the maturation status of myofibers (Fig. 3d). To confirm the cytoplasmic accumulation, subcellular fractionation assays were performed on myoblasts and myotubes, and corroborated the immunofluorescence findings: NuMA1 was exclusively nuclear in proliferative myoblasts, but present in both nuclear and cytoplasmic fractions in myotubes (Fig. 3e, f). Similarly, differentiated human primary myoblasts exhibited a progressive cytoplasmic accumulation of NuMA1, confirmed by fractionation analysis (Fig. 3g and Supplementary Fig. 3f). We next investigated NuMA1 subcellular localization in long-term mature myofibers using *ex-vivo Tibialis Anterior* (TA) myofibers (Fig. 3h). Immunostainings revealed predominant nuclear NuMA1, with a cytoplasmic pool capping the extremities of elongated myonuclei. These regions overlapped with MTs and were preferentially situated at microtubule nucleation sites in mature myofibers [40]. Together, these results demonstrate that NuMA1 is exclusively nuclear in proliferating murine and human myoblasts but progressively accumulates in the cytoplasm upon muscle cell differentiation, with distinct spatial enrichment in mature myofibers.

To better understand the role of cytoplasmic NuMA1 in myofibers, we overexpressed in myofibers either wild-type NuMA1 (i.e., NuMA1-WT), containing the NLS sequence, or a construct in which the NLS sequence is replaced by a NES sequence [41], allowing NuMA1 to be restrained into the cytoplasmic compartment (i.e., NuMA1-NES). Immunofluorescence analysis revealed that NuMA1-WT, as expected, accumulated into the nucleus compartment while NuMA1-NES showed a membrane-like structure, either as short dots in cells or as elongated structures, dispersed all along the length of myofibers (Supplementary Fig. 3g). To note, these dense NuMA1-NES membrane-like structures do not colocalize with PCM1 and are devoid of MTs, resulting in local myofibers’ cytoplasmic areas deprived of MTs (Supplementary Fig. 3g).

We next tracked myonuclei displacement within myofibers expressing either NuMA1-WT or NuMA1-NES. As overexpression of NuMA1-WT leads to a mosaic regarding NuMA1 myonuclei accumulation, we used this difference in NuMA1 myonuclei accumulation to categorize myonuclei according to NuMA1 content (low (WTL) *versus* high (WTH) content) and correlated nuclear NuMA1 levels to myonuclei motility. This approach shows that low content of NuMA1 inside myonuclei correlates with a lower myonuclei motility, reflected by a 40% drop in the total distance traveled by myonuclei inside myofibers, mainly attributed to the decrease in the percentage of time myonuclei are in motion, while high NuMA1 myonuclei content correlates with enhanced time in motion and displacement of myonuclei (Supplementary Fig. 3h–j). Analysis of myonuclei in myofibers expressing NuMA1-NES shows a significant decrease in the percentage of time in which myonuclei are moving inside myofibers (more than 50%), that *in fine* leads to a 20% decrease in the total distance traveled by each myonuclei inside myofibers (Supplementary Fig. 3h–j). Collectively, our results show a positive correlation between myonuclei NuMA1 content and myonuclei velocity and point to a cytoplasmic role for NuMA1, inducing the formation of membrane-like structures that impede myonuclei displacement through the formation of MT-devoid territories.

### NuMA1 controls the microtubule network throught nuclear-MTOC organization

**Fig. 4:**
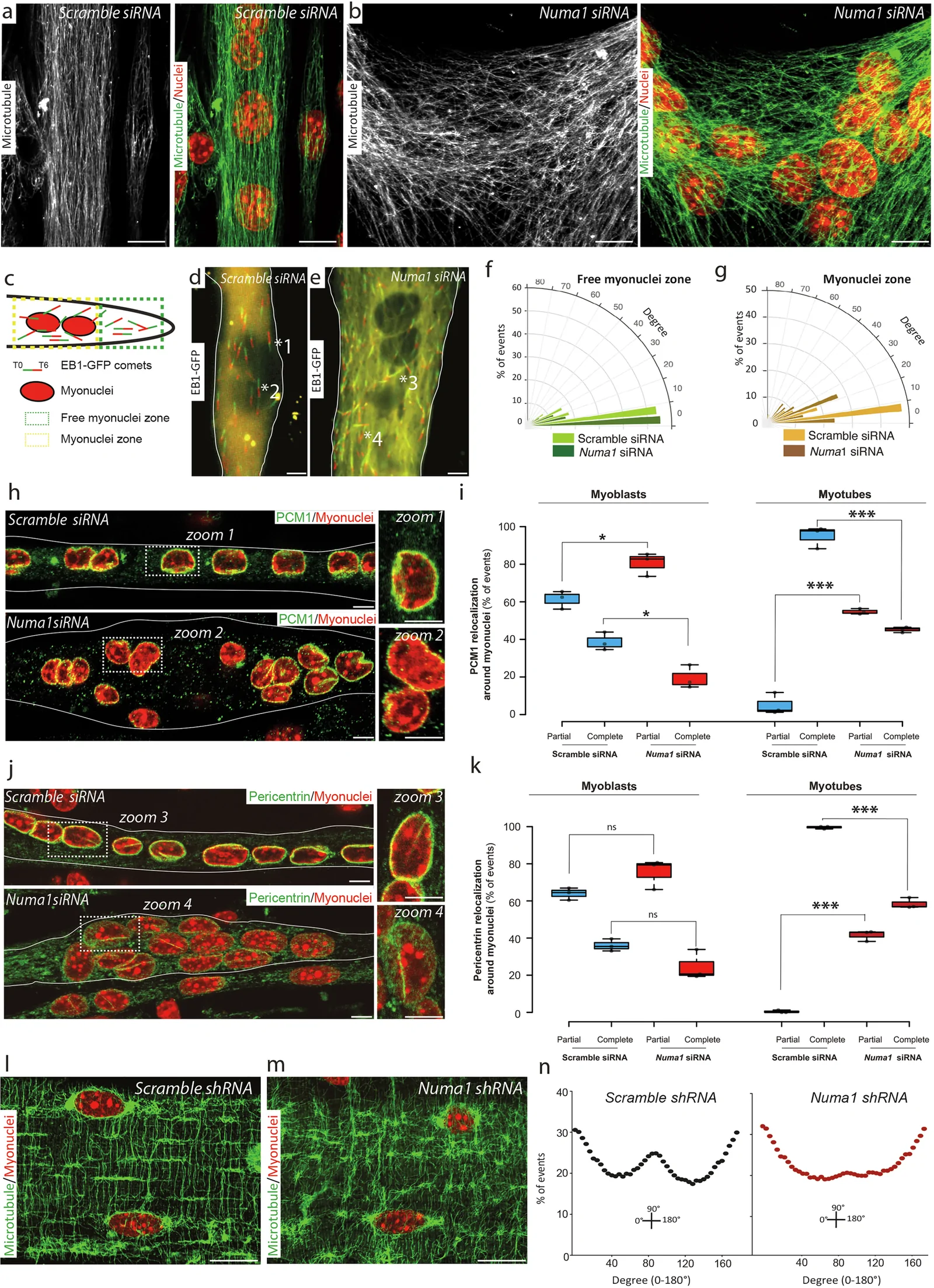
NuMA1 shapes MT network and nMTOC architecture in myofibers. **a, b** Representative images of 3-day primary myotubes transfected with scramble (**a**) or *Numa1* (**b**) siRNA, stained for myonuclei (red) and microtubules (green) in vitro (Scale bar: 10 µm). **c** Schematic of EB1-GFP comets analysis in 3-day primary myotubes. **d, e** Representative maximal Z-projections of EB3-GFP comets in 3-day primary myotubes after scramble (**d**) or *Numa1* (**e**) siRNA transfection, where six consecutive frames were overlapped (first three in green, last three in red) (Scale bar: 9 µm). White asterisks indicate comet orientation in control (d-1 & 2) and in *Numa1* knockdown condition (e-3 & 4). **f, g** Quantification of EB1-GFP comets orientations along the myotube axis at tips (*n* = 3 independent primary cultures, 175 analyzed events in scramble, 260 in *Numa1* knockdown) (**f**), and in myonuclei areas (*n* = 3 independent primary cultures, 284 analyzed events in scramble siRNA-, 376 in *Numa1* siRNA-transfected) (**g**). Only myotubes with ≥6 myonuclei were analyzed. **h** Representative images of 3-day primary myotubes after scramble or *Numa1* siRNA transfection, stained for myonuclei (red) and Pericentrin (PCM1, green) in vitro (Scale bar: 10 µm)**. i** Quantification of the percentage of myonuclei with partial or complete PCM1 relocalization in primary myoblasts and 3-day primary myotubes after scramble or *Numa1* siRNA transfection (*n* = 3 independent primary cultures). **j** Representative images of 3-day primary myotubes after scramble or *Numa1* siRNA transfection stained for myonuclei (red) and Pericentrin (green) in vitro (Scale bar: 10 µm)**. k** Quantification of the percentage of myonuclei with partial or complete Pericentrin relocalization around myonuclei in primary myoblasts and 3-day primary myotubes after scramble or *Numa1* siRNA transfection (*n* = 3 independent primary cultures). **l**, **m** Representative images of *Tibialis Anterior* isolated myofibers from 2-month-old WT mice after 2 weeks of scramble (**l**) or *Numa1* (**m**) shRNA down-regulation, stained for myonuclei (red) and microtubules (green) (Scale bar: 10 µm). **n** Microtubule network organization in *Tibialis Anterior* isolated myofibers from 2-month-old WT mice after 2 weeks of scramble or *Numa1* shRNA, analyzed with TeDT software (*n* = 5 mice/condition). Graphical boxes showed the total data population represented by the higher and the lower point of the population. The median is represented. Unpaired T-test was performed between groups. Each *p*-value is mentioned in the figure legend of each graph, ns (not significant) *p < 0.05, **p < 0.01 and ***p < 0.001.

NuMA1 contains two MT-binding domains that play a crucial role during mitosis, especially for MT nucleation and mitotic spindle orientation [42–45]. As myonuclei alignment in myotubes is mainly mediated by MT interactors [5], we next investigated the role of NuMA1 in maintaining MT network architecture during muscle differentiation (Fig. 4). We first examined MT network organization in *Numa1*-depleted myotubes using immunostaining. As expected, depletion resulted in numerous, non-aligned MT intersections compared with the well-ordered arrays observed in scramble conditions (Fig. 4a, b). To better identify the cause of this MT disorganization, we analyzed MT plus-end dynamics and orientation using live-cell microscopy of GFP-tagged End-Binding-protein-1 (EB1) in either scramble or *Numa1* siRNA conditions. EB1-GFP comets velocity was unaffected by *Numa1* depletion, suggesting that NuMA1 does not regulate MT polymerization rate (Supplementary Fig. 4a, b). As we previously observed cytoplasmic NuMA1 adjacent to the myonuclei envelope in mature myofibers (Fig. 3h), we next analyzed MT plus-end orientation in two defined regions of myotubes: the “myonuclear zone” and the “nuclear-free zone” (distal tips of myotubes) (Fig. 4c). In control conditions, MT growing ends orientation are mainly tangential to the longitudinal axis of myotubes, no matter the presence of myonuclei (Fig. 4c–g). Interestingly, in *Numa1* depleted conditions, EB1-GFP comets trajectories appear with more randomized orientations in the “myonuclei zone” while the orientation was not affected in the “free myonuclei zone” (Fig. 4f, g). This result suggests a specific role of NuMA1 at the vicinity of myonuclei rather than all along myotubes length. In myotubes, MTs mainly emanate from the myonuclei membrane (i.e., nMTOC) thanks to the sequential recruitment of several MT nucleator proteins at the membrane of myonuclei, such as AKAP9, Pericentrin, and the Pericentriolar Material 1 (PCM1) [5]. NuMA1 was previously identified as a MT minus-end interactor in mitosis [45]. We thus examined the recruitment of components of the nMTOC throughout the differentiation process in *Numa1*-depleted conditions in both myoblasts and myotubes (Fig. 4h–k). In control conditions, differentiating myoblasts progressively accumulate at myonuclei membrane PCM1, Pericentrin, and Golgi proteins. This process is usually partial in myocytes and fully completed in myotubes, reflected by a 100% coverage of the staining for these proteins with the membrane of myonuclei (Fig. 4h–k and Supplementary Fig. 4c–e). Interestingly, in *Numa1*-depleted conditions, we observe a failure in the ability of myoblasts to recruit PCM1 at the nuclear membrane but no significant changes in the recruitment of Pericentrin or Golgi proteins (Fig. 4h–k and Supplementary Fig. 4c–e). However, in control myotubes, 100% of myonuclei exhibit PCM1, Pericentrin, and Golgi proteins at the vicinity of myonuclei while in *Numa1*-depleted conditions, only 58% of myonuclei present a full recruitment of PCM1, 45% of myonuclei present a full recruitment of Pericentrin and 42% of myonuclei present a full recruitment of Golgi proteins (Fig. 4h–k and Supplementary Fig. 4c–e). These results suggest that cytoplasmic-NuMA1, through the control of proteins recruitment at the nMTOC, constrains MT growth orientation in the longitudinal axis of myotubes. To confirm in vivo the key role of NuMA1 in the organization of the microtubules network, we conducted *Numa1* depletion in vivo for 2 weeks in the TA muscle of 8-week-old mice (Fig. 4l, m). Using this approach, we observed a strong alteration of the MT network architecture with an apparent loss of transversally oriented MTs compared to longitudinally oriented MTs observed in control (Fig. 4l–n). To better appreciate the impact of this MTs alteration on myonuclei behavior, we quantified myonuclei localization in shRNA-positive myofibers from TA transversal cross-sections. This analysis shows a five-fold increase in mispositioned myonuclei compared to control condition which appear internalized rather than at the periphery or at the center of myofibers (Supplementary Fig. 4f–h). Additionally, the distribution of cross-sectional area of TA myofibers depleted of NuMA1 appears smaller, suggesting a reduction of MND in *Numa1*-depleted myofibers (Supplementary Fig. 4i). Altogether, our data shows that the cytoplasmic NuMA1 in developing myofibers shapes the MT patterning through the control of myonuclei-MT anchoring points contributing to myonuclei displacement and positioning.

### NuMA1 accumulation in the nucleus is controlled by AMPKɑ1 activity

**Fig. 5:**
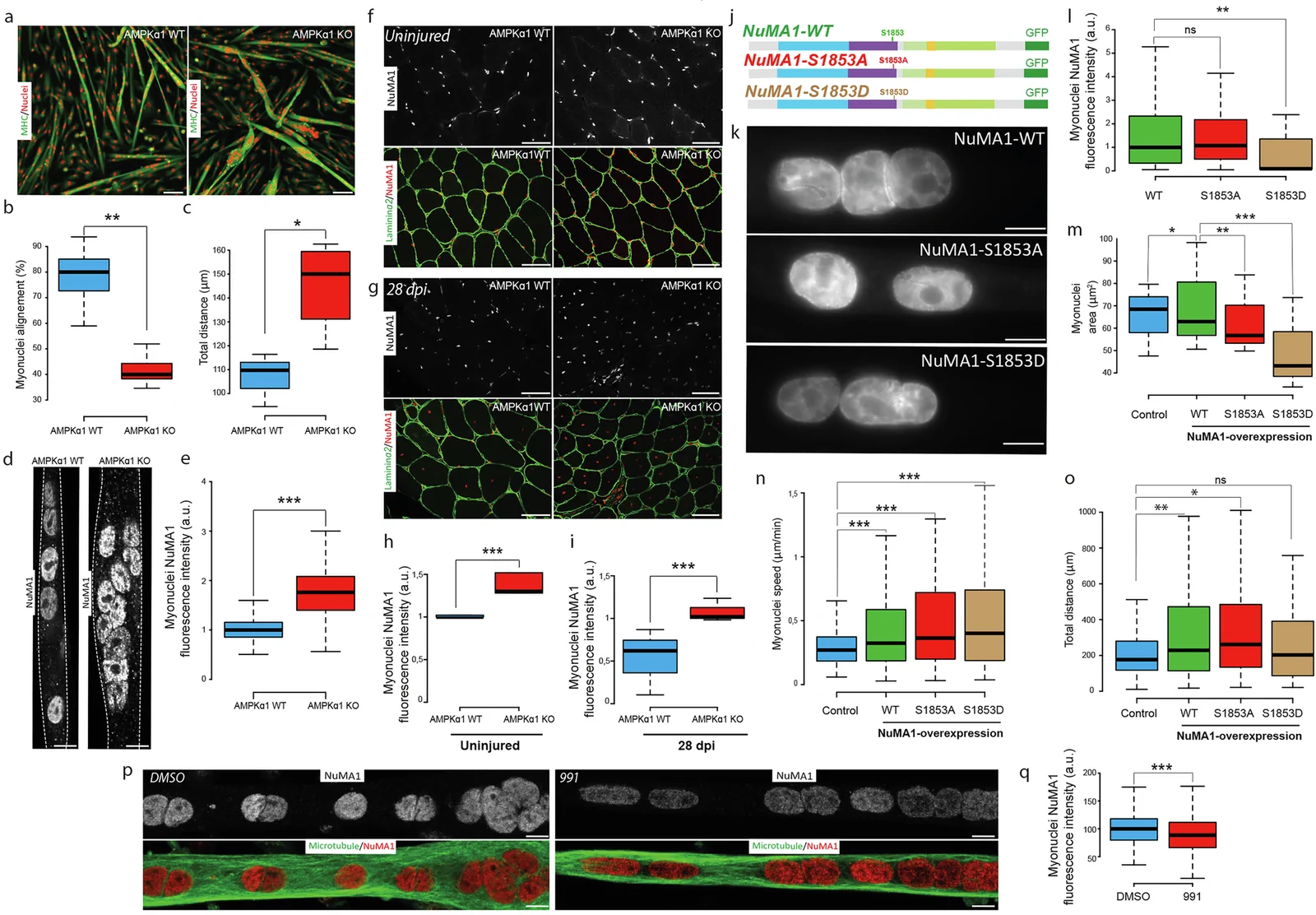
AMPKɑ1 regulates NuMA1 localization through phosphorylation. **a** Representative images of AMPKɑ1 WT or KO 3-day primary myotubes stained for Myosin Heavy Chain (MHC, green) and nuclei (red) in vitro (Scale bar: 50 µm). **b** Quantification of the percentage of AMPKɑ1 WT or KO 3-day primary myotubes with aligned myonuclei (*n* = 3 independent primary cultures, 24 mosaic images in WT and 27 in KO). **c** Quantification of the total distance (µm) traveled by myonuclei in AMPKɑ1 WT or KO 5-day primary myofibers (*n* = 3 independent WT cultures with 536 myonuclei analyzed and *n* = 4 independent KO primary culture with 505 myonuclei tracked). **d** Representative images of AMPKɑ1 WT or KO 3-day primary myotubes, stained for NuMA1 (gray) (Scale bar: 10 µm). **e** Quantification of nuclear NuMA1 intensity (a.u.) in AMPKɑ1 WT or KO 3-day primary myotubes (*n* = 3 independent primary cultures, 234 WT and 278 KO myonuclei analyzed). **f** Representative images of *Tibialis Anterior* cross-sections from 2-month-old AMPKɑ1 WT or KO mice without injury, stained for NuMA1 (red) and lamininɑ2 (green) (Scale bar: 50 µm). **g** Representative images of *Tibialis Anterior* cross-sections from 2-month-old Pax7-CreER^T2/+^; AMPKɑ1^fl/fl^ mice 28 days post-injury (dpi) with cardiotoxin, stained for NuMA1 (red) and lamininɑ2 (green) (Scale bar: 50 µm). **h** Quantification of NuMA1 intensity (a.u.) in myonuclei of uninjured *Tibialis Anterior* cross-sections from 2-month-old AMPKɑ1 WT or KO mice (*n* = 3 mice/condition, 88 WT myonuclei and 173 KO myonuclei analyzed). **i** Quantification of NuMA1 intensity (a.u.) in myonuclei of *Tibialis Anterior* cross-sections of 2-month-old Pax7-CreER^T2/+^ (AMPKɑ-WT) or *Ampkɑ1*^*fl/fl*^- Pax7-CreER^T2/+^ (AMPKɑ-KO) mice, 28 days post-injury (dpi) from cardiotoxin injection (130 WT myonuclei and 160 KO myonuclei analyzed). **j** Scheme of NuMA1-WT protein domains. The second construct corresponds to a non-phosphorylable mutant in which serine-1853 is substituted by alanine (S1853A), while the third corresponds to a phosphomimetic mutant in which serine-1853 is replaced by aspartic acid (S1853D). **k** Representative images of 3-day primary myotubes transfected with NuMA1-WT, NuMA1-S1853A or NuMA1-S1853D plasmids, identified with plasmid-GFP (gray) (Scale bar: 10 µm). **l** Quantification of nuclear NuMA1 intensity (a.u.) in 3-day primary myotubes after overexpression of NuMA1-WT, NuMA1-S1853A or NuMA1-S1853D (*n* = 3 independent primary cultures, 142 WT myonuclei, 119 S1853A, and 81 S1853D analyzed). **m** Quantification of myonuclear area (µm^2^) in control 3-day primary myotubes or after overexpression of NuMA1-WT, NuMA1-S1853A, and NuMA1-S1853D (*n* = 3 independent primary cultures, 281 control myonuclei, 176 WT, 209 S1853A, and 124 S1853D analyzed). **n, o** Quantification of myonuclear speed (µm/min) (**n**) and total distance traveled by myonuclei (**o**) in control 5-day primary myofibers or after overexpression of NuMA1-WT, NuMA1-S1853A, and NuMA1-S1853D (*n* = 2 independent primary control cultures, 215 myonuclei tracked; *n* = 3 independent primary cultures, 149 WT, 121 S1853A, and 109 S1853D myonuclei tracked). **p** Representative images of WT 3-day primary myotubes treated with either DMSO or 991, stained for NuMA1 (red) and microtubules (green) (Scale bar: 10 µm). **q** Quantification of nuclear NuMA1 intensity (a.u.) in WT 3-day primary myotubes treated with either DMSO or 991. Graphical boxes showed the total data population represented by the higher and the lower point of the population. The median is represented. Unpaired T-test was performed between groups. Each *p*-value is mentioned in the figure legend of each graph, ns (not significant) *p < 0.05, **p < 0.01 and ***p < 0.001.

As numerous MAPs, NuMA1 activity is controlled by phosphorylation [46]. Several kinases have been identified targeting NuMA1 to control the mitotic spindle orientation during mitosis such as ABL1, Aurora-A, Plk1 or CDK1 [47–49] (Supplementary Fig. 5a). In a biochemical approach, Schaffer *et al* identified NuMA1 as a direct target of AMPK [50] with AMPK phosphorylation site localized on the serine-1853, in the C-terminus part of NuMA1 (Supplementary Fig. 5a). AMPK activity in muscle stem cells is mainly assigned to AMPKɑ1 rather than AMPKɑ2 [51]. To confirm the implication of AMPK in myofiber formation, we thus first investigated myonuclei organization and velocity in primary myotubes using either WT or AMPKɑ1*-*KO myotubes (Fig. 5a–c). AMPKɑ1*-*KO myotubes exhibit twice less myotubes with aligned myonuclei (Fig. 5a, b), unrelated to the alteration in myoblast fusion (Supplementary Fig. 5b) showing that AMPK, as NuMA1, is involved in setting MND in developing myotubes (Fig. 2). Quantification of myonuclei velocity did not reveal any significant alteration (Supplementary Fig. 5c). However, the total distance displacement was enhanced by more than 30%, passing from 110 µm in WT condition to 150 µm in AMPKɑ1 KO myotubes (Fig. 5c) and was mainly attributed to a decrease in the time myonuclei “pause” in myotubes (Supplementary Fig. 5d).

These data show that AMPKɑ1 activity is required for myonuclei spreading in myotubes and suggest that NuMA1-AMPKɑ1-related phosphorylation restrain myonuclei displacement, potentially through the maintenance of a cytoplasmic pool of NuMA1 in myotubes. We thus investigated if the absence of AMPKɑ1 impacts nuclear accumulation of NuMA1 in AMPKɑ1*-*KO myotubes/myofibers (Fig. 5d–i). We first analyzed in AMPKɑ1-KO myotubes, nuclear NuMA1 content using immunofluorescence approaches (Fig. 5d). Quantification of the NuMA1 mean intensity in myonuclei shows a 70% increase in AMPKɑ1-KO myotubes compared to control condition (Fig. 5e). To confirm this results, we investigated on the nuclear NuMA1 intensity in myofibers using transverse sections of 2 months-old TA muscle fibers, extracted from either WT or AMPKɑ1*-*KO mouse (Fig. 5f). Nuclear NuMA1 intensity quantification shows an increase of 30% in AMPKɑ1 KO myofibers compared to control (Fig. 5h). To avoid technical variations due to the localization of myonuclei in mature myofibers (at the periphery or under the basal lamina), we performed a TA muscle injury step in either WT or AMPKɑ1 KO mice using cardiotoxin injection that induced a complete muscle regeneration process. In these conditions, after 28 days, all myonuclei remain in the center of myofibers [5, 52], allowing a better discrimination of myonuclei (Fig. 5g). Quantification of NuMA1 myonuclei intensity shows 40% increase in the mean intensity (Fig. 5i), confirming that AMPKɑ1 has a role in the accumulation of NuMA1 in the nuclear compartment. To assess if NuMA1 phosphorylation status is necessary for this preferential accumulation, we overexpressed mutated version of human NuMA1, tagged with GFP and mimicking either a non-phosphorylable version of NuMA1 (with serine-1853 replaced by an alanine) or a phospho-mimetic version of NuMA1 (with serine-1853 replaced by an aspartic acid) (Fig. 5j). As expected, the human NuMA1-GFP constructs exhibit a myonuclei staining, with various accumulation of NuMA1 inside myonuclei, suggesting intrinsic variability in the NuMA1 protein uptake by myonuclei according to the phosphorylation status (Fig. 5k and Supplementary Fig. 5e). Quantification of NuMA1 mean intensity into myonuclei shows no differences between NuMA1-WT and the non-phosphorylable version of NuMA1 while it decreases about half in the phospho-mimetic version of NuMA1 (Fig. 5l), confirming that the phosphorylation status affects its ability to be kept by myonuclei. Interestingly, NuMA1 overexpression affects myonuclear shape, resulting in smaller nuclei, as reflected by a decrease in myonuclei area (Fig. 5m). We next addressed whether the phosphorylation status affects myonuclear speed and displacement within myofibers (Fig. 5n, o and Supplementary Fig. 5f). We found that overexpression of all NuMA1 constructs correlates with increased myonuclei speed and displacement (Fig. 5n, o). To note, the phospho-mimetic version of NuMA1 exhibits threefold more pausing compared to controls, NuMA1-WT and the non-phosphorylable version of NuMA1, which may explain why the total distance traveled by myonuclei remains unchanged relative to control conditions (Fig. 5o and Supplementary Fig. 5f). These data suggest that nuclear NuMA1 content can potentially reflect the motility of myonuclei and that the AMPK-related phosphorylation of NuMA1 restrain its uptake by myonuclei and block the movement by inducing myonuclei pauses. Finally, to better show that the activity of AMPK controls the ability of myonuclei to uptake NuMA1, we used Benzimidazole derivative small-molecule 991, a known enhancer of AMPK activity [53] and applied it directly onto WT primary myotubes (Fig. 5p, q). Our results show that the activation of AMPK leads to the decrease of the mean intensity of NuMA1 inside myonuclei after 1 h treatment (Fig. 5q), suggesting that the myonuclei uptake control of NuMA1 is a dynamic process. Together, our data show that AMPK, through its phosphorylation activity on the serine-1853 of NuMA1, controls its uptake by myonuclei and impacts myonuclei velocity and shape.

### NuMA1 is accumulated in dystrophic myonuclei and tunable by drugs targeting AMPK activity

**Fig. 6:**
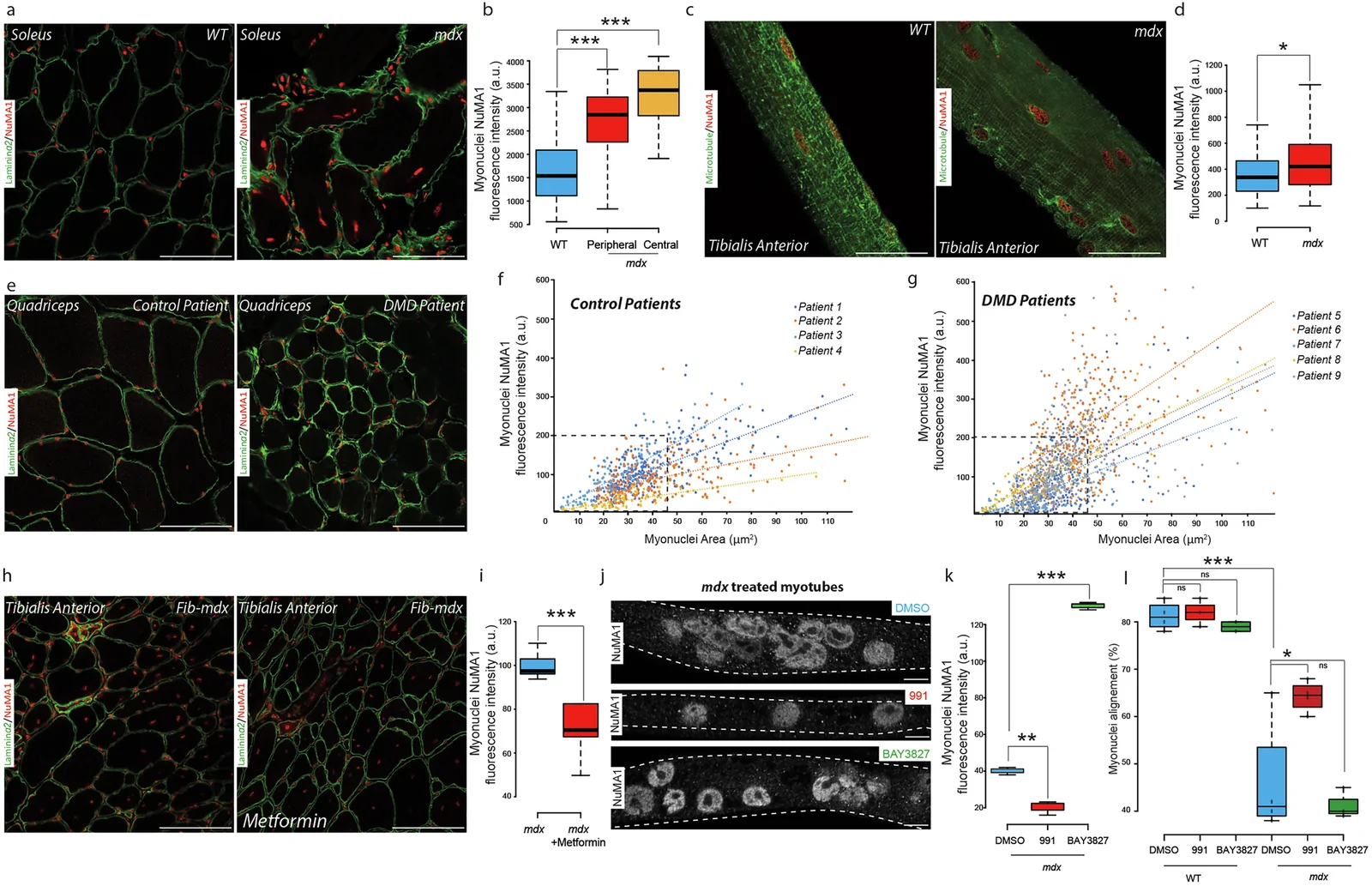
NuMA1 in myonuclei of Duchenne muscular dystrophy. **a** Representative images of *Soleus* cross-sections from 2-month-old WT or *mdx* mice, stained for NuMA1 (red) and lamininɑ2 (green) (Scale bar: 150 µm). **b** Quantification of NuMA1 intensity (a.u.) in peripheral and centralized myonuclei of *Soleus* cross-sections from 2-month-old WT or *mdx* mice (*n* = 3 mice/condition). **c** Representative immunofluorescence images of isolated *Tibialis Anterior* myofibers from 2-month-old WT or *mdx* mice, stained for microtubules (green) and NuMA1 (red) (Scale bar: 25 µm). **d** Quantification of NuMA1 intensity (a.u.) in myonuclei of *Tibialis Anterior* isolated myofiber from 2-month-old WT or *mdx* mice (*n* = 3 mice/condition, 188 WT and 295 *mdx* myonuclei analyzed). **e** Representative images of *quadriceps* cross-sections from control and DMD patient biopsies, stained for NuMA1 (red) and lamininɑ2 (green) (Scale bar: 150 µm). **f, g** Quantification of NuMA1 intensity (a.u.) relative to myonuclei area (µm^2^) in control (**f**) or DMD (**g**) patient biopsies (*n* = 4 control patients, 270 analyzed myonuclei for patient 1, 259 for patient 2, 127 for patient 3 and 68 for patient 4. *n* = 5 DMD patients, 346 analyzed myonuclei for patient 5, 377 for patient 6, 117 for patient 7, 109 for patient 8 and 430 for patient 9). **h** Representative images of *Tibialis Anterior* cross-sections from 3-month-old *mdx* mice treated or not with metformin, stained for NuMA1 (red) and lamininɑ2 (green) (Scale bar: 150 µm). **i** Quantification of NuMA1 intensity (a.u.) in myonuclei of *Tibialis Anterior* cross-sections from 3-month-old *mdx* mice treated or not with metformin (*n* = 5 mice/condition, 545 analyzed myonuclei in untreated *mdx* and 488 after metformin treatment). **j** Representative images of 3-day *mdx* primary myotubes treated with DMSO, 991, or BAY3827 and stained for NuMA1 (gray) (Scale bar: 10 µm). **k** Quantification of NuMA1 intensity (a.u.) in myonuclei in 3-day *mdx* primary myotubes treated with DMSO, 991 or BAY3827 (*n* = 3 independent primary cultures, 2029 DMSO, 111 in 991 and 2668 in BAY3827 analyzed myonuclei). **l** Quantification of the percentage of *mdx* myotubes with aligned myonuclei after DMSO, 991 or BAY3827 treatment (*n* = 4 independent primary cultures for DMSO; *n* = 3 independent primary cultures 991 and BAY3827 treatments). Graphical boxes showed the total data population represented by the higher and the lower point of the population. The median is represented. Unpaired T-test was performed between groups. Each *p*-value is mentioned in the figure legend of each graph, ns (not significant) *p < 0.05, **p < 0.01 and ***p < 0.001.

Our finding shows that myonuclei with increased mobility accumulate NuMA1 inside myonuclei (Fig. 5) and that *mdx* myonuclei are highly dynamic myonuclei (Fig. 1) suggesting a correlation between NuMA1 myonuclei content and their ability to move inside myofibers. To further confirm this hypothesis, we first quantified NuMA1 intensity in myonuclei from 2-months old WT or *mdx* mice, using transversal muscle section of *Soleus* muscle (Fig. 6a, b). We showed that *mdx* myofibers exhibit increased NuMA1 myonuclei content, associated with reduced cytoplasmic NuMA1 intensity, suggesting a failure in NuMA1 content equilibrium between myonuclei and cytoplasm (Fig. 6b and Supplementary Fig. 5g). Interestingly, peripheral myonuclei accumulated less NuMA1 than centralized myonuclei, suggesting different NuMA1 uptake dynamics in peripheral compared to centralized myonuclei (Fig. 6b). We confirmed these results using extracted myofibers from *mdx* TA muscle (Fig. 6c, d). We next addressed whether proteins known to re-localize around myonuclei are affected under these conditions, such as Pericentrin or Golgi apparatus. We found that in *mdx* myofibers, Pericentrin still localizes around myonuclei; however, because myonuclei are more rounded, the “polarized” Pericentrin localization is lost (i.e., at the two extremities of myonuclei along the longitudinal axis) (Supplementary Fig. 6a). Interestingly, Golgi apparatus localization, appearing as longitudinal puncta distributed along the longitudinal axis, seems preserved (Supplementary Fig. 6b). To explore our results in human pathological context, we next obtained *Quadriceps* biopsies from either control or DMD patient to performed quantification of NuMA1 myonuclei intensity on transversal muscle sections (Fig. 6e–g). With this approach, we first observed a strong heterogeneity in NuMA1 intensity, nevertheless when NuMA1 intensity is plotted according to myonuclei area, we found that in control patients, 72% of myonuclei exhibit a level of NuMA1 that do not excess 2-fold change (with myonuclei area limited to 45 µm^2^) (Fig. 6f). On the opposite, this category of myonuclei only reach 46% in DMD patients, with some myonuclei that accumulate up to 6-fold NuMA1 in myonuclei with no particular change in myonuclei area distribution (Fig. 6g). These data confirm a common pathway in Duchenne myopathy regarding the accumulation of NuMA1 in nuclear compartment and suggest a variability in the motility of myonuclei inside myofibers. Finally, based on our previous finding that activation of AMPK pathway with the Benzimidazole derivative small-molecule 991 inhibited NuMA1 myonuclei accumulation (Fig. 5p, q) and the reported ability of AMPK stimulation to enhance muscle function and protect muscle in dystrophic mice [54–56], we next explored whether treatment of *mdx* mice with an AMPK activator would influence nuclear NuMA1 accumulation (Fig. 6h, i). We thus evaluated in fibrotic dystrophic mice model [57] the effect of metformin, a known activator of the AMPK pathway [58]. Our results showed that metformin-related activation of AMPK results in a 30% decrease in the mean intensity of NuMA1 inside myonuclei (Fig. 6h, i), suggesting that the benefit of metformin treatment in *mdx* mice models could be partly due to its effect on NuMA1 myonuclei shuttling [54]. To better appreciate the direct implication of the AMPK activity effect in *mdx* muscle cells, we next manipulated the activity of AMPK by treating *mdx* myotubes either with a specific activator of AMPK (Benzimidazole derivative small-molecule 991) or with an inhibitor of the AMPK (BAY 3827 [59]) (Fig. 6j–l). Activation of the AMPK pathway, as expected, decreased the intensity of nuclear NuMA1 (correlated with an increase in the cytoplasmic compartment) while the inhibition dramatically enhanced nuclear NuMA1 (correlated with a decrease in the cytoplasmic compartment) (Fig. 6j, k and Supplementary Fig. 5h). Our findings also showed that AMPK activity restores the capacity of myonuclei to correctly align in developing myofibers (Fig. 6l). Interestingly, we did not observe any effect with the activation or inhibition of the AMPK pathway in control conditions while we observed a clear benefit of the AMPK pathway activation in *mdx* myotubes with a 60% increase in the ability of myotubes to correctly align myonuclei (Fig. 6l). Our observation thus shows that in dystrophic condition, NuMA1 shuttling between the cytoplasmic compartments and myonuclei is controlled by the AMPK activity and can be pharmacologically modulated with a benefic effect on MND patterning.

## Discussion

Myonuclei are located between the plasma membrane of myofibers and myofibril structures [60]. Their peripheral localization induces drastic changes in their shape, primarily due to forces exerted on the nuclear envelope. This conformational adaptation is thought to stabilize both internal and external mechanical forces, and consequently, to influence chromatin organization and gene expression [17, 61]. The spatial organization of peripheral myonuclei is determined by the interplay among various cytoskeletal components (microtubule, actin and intermediate filaments). Consequently, myonuclei spatial distribution establishes MND within myofibers, which contribute to the spatial coordination of transcriptomic activity, and, ultimately, to the functional integrity of the myofiber [62]. Accordingly, nuclear spreading at the periphery of muscle fibers is a prerequisite to obtain fully functional mature myofibers [1, 3, 8]. Myonuclei are considered relatively stable when peripheral, remaining anchored at specific locations along mature mammalian skeletal muscle fibers. However, it is common to observe merged/aggregated myonuclei with various shapes and orientations along mature myofibers, particularly in aged myofibers [27], suggesting that myonuclei possess varying abilities to move within the same myofiber.

### Myonuclei motility is a marker of pathological conditions and is linked to the microtubule nucleation at the myonuclei membrane

In this study, we show that the modulation of an optimal myonuclei motion is deleterious for the establishment of MND settings in developing myofibers. Decreasing the ability of myonuclei to move during the first steps of myotube formation dramatically impaired the initial steps for myonuclei spreading, leading to myonuclei aggregation that counteracts the correct establishment of MND settings [1, 8]. In pathological conditions such as DMD, enhanced myonuclei motility is a precocious event as it can be detected in the first steps of myotube formation (Fig. 1) and persists as a long-term phenotype in *mdx* mice model [63]. Increased myonuclei motility also induces myonuclei aggregation, suggesting that an “optimal” myonuclei motility is needed to set the fundaments of MND in developing myofibers. Interestingly, myonuclei shape (i.e., roundness, area) can not be considered as a motility marker as we observed in *mdx* and *Numa1* depleted myofibers, opposite effects on myonuclei velocity while both conditions increased myonuclei circularity (Figs. 1, 2). NuMA1, as a soft space-filling or matrix protein, could three-dimensionally scaffold other nucleoskeletal structures and its depletion could mediate a new repartition of key proteins of the LINC complex, such as Nesprin family members, that sustain the interaction with the MTs network [11, 13, 14, 64]. Interestingly, in DMD conditions, proteins already identified to control myonuclei motility, such as Kif5b or MAP7 are dramatically reduced, impacting the pool of interactors available for myonuclei displacement [63]. All in all, the precise mapping of motility factors present inside and outside myonuclei will be more informative to predict myonuclei motility rather than “shape status” extrapolation. It is also important to note that myonuclei displacement is not primarily driven by the “speed capacity” of myonuclei but rather by their ability to enchain periods of movements, that are controlled by a large family of molecular motors in muscle cells [23]. In this view, cytoplasmic NuMA1 could act on these movements by regulating the availability/localization of motors responsible for movement, with Dynein family members representing strong candidates [65].

Here, we elucidate the mechanisms by which myofibers control myonuclei motion during muscle fiber formation. Indeed, we demonstrate that the MT binding protein NuMA1 is present at once in the nucleus and in the cytoplasm of developing myofibers (Fig. 3). Cytoplasmic NuMA1 acts on myonuclei motion abilities through the modulation of the cytoskeleton meshwork in the vicinity of myonuclei and controls nMTOC proteins recruitment at the membrane of myonuclei (Fig. 4). Interestingly, this activity is regulated by AMPK phosphorylation, which contributes to restrain NuMA1 inside the cytoplasmic compartment and thus counteracts the capacity of NuMA1 to be taken up by nuclei through its NLS domain, leading to the presence of myonuclei with varying NuMA1 content, correlated with their motility ability (Fig. 5 and Supplementary Fig. 3). The mechanism contributing to the cytoplasmic localization of NuMA1 is still pending. Alteration in nuclear shape or increase of nuclear volume during differentiation/maturation of myofibers could increase tension in the nuclear envelope, which could in turn trigger nuclear pore complex opening and the release of NuMA1 from nuclei [66]. Recently, NuMA1 was shown to act as a dynein adaptor to connect it to the MTs minus-end [65]. The authors show that NuMA1 binds directly and preferentially to the MTs minus-ends, stopping their growth assembly and protecting MTs against shrinkage but their results show that this process needs γ-TuRC uncapping, suggesting that NuMA1 can only associate with uncapped γ-TuRC MTs or severed MTs. In this view, MTs severing events could favor the accumulation of NuMA1 in the cytoplasm in the course of myofiber formation. Finally, our experiments show that in myofibers, NuMA1 can form membrane-like structures, possibly through the direct association with phosphoinositides [67] or through its binding with the membrane-anchored protein LGN, that tether dynein to the cell cortex [68]. This cytoplasmic function could contribute to its retention in the cytoplasmic compartment but also favor particular MT architecture observed in skeletal muscle cells, strongly affected upon *Numa1* depletion (Fig. 4 and Supplementary Fig. 3g). It is tempting to think that in the course of myotube formation, after fusion events, the variable motility of myonuclei observed is dependent on existing NuMA1 content in myoblasts’ myonuclei, and over time, NuMA1 uptake by myonuclei becomes progressively more homogenous throughout the length of myotubes/myofibers allowing a “myonuclei velocity equilibrium” that favors myonuclei spreading. This hypothesis is supported by the strong differences in myonuclei content between myotubes and myofibers (Fig. 3g). Future work should address the status of NuMA1 in myonuclei content during accretion phases, as well as the local activity of AMPK that spatially controls myonuclei spreading.

### A key role for NuMA1 in skeletal muscle fibers

In proliferating cells, NuMA1 is a nuclear protein, playing an important role in chromosome decondensation at the mitotic exit [69]. During mitosis, NuMA1 interacts with dynein to reach the poles of mitotic spindles to stabilize and oriente them [70]. At the end of mitosis, the nuclear envelope is reformed, NuMA1 is then taken up inside the nucleus with its NLS sequence [71]. NuMA1 was initially described to be degraded in post-mitotic muscle cells [72]. In our study, we demonstrate that NuMA1 is well expressed in developing myofibers (Fig. 3) and we discover a cytoplasmic role of this protein in the post-mitotic myofibers (Figs. 3–5). Indeed, we show that NuMA1 controls nMTOC proteins recruitment at the membrane of myonuclei allowing the correct MT network polymerization orientation inside the developing myofibers to permit MND establishment (Figs. 2–4). NuMA1 was recently identified to have a cytoplasmic role in post-mitotic neurons, in the context of Huntington’s disease, also with an impact on the MT network within the growth cone of the axon [73]. Despite the well-documented cytoplasmic role of NuMA1 in mitosis, its nuclear role remains elusive. NuMA1 is a large coiled-coil protein present throughout the nucleus [74] and is nearly as abundant as nuclear lamins, suggesting a major role as a component of the nucleoskeleton [39]. In this view, NuMA1 has the capacity to form long coiled-coil homodimers that can self-assemble to generate three-dimensional space-filling structures [75], likely contributing to nuclear shape. Interestingly, we found that in *Numa1*-depleted muscle cells, myonuclei circularity is increased in myotubes (Fig. 2), and NuMA1’s phosphorylation status also impacts myonuclei conformation (Fig. 5). Additionally, NuMA1’s involvement in chromatin and nuclear architecture organization is associated with cellular differentiation [76], and consequently could mediate genome organization and mechanotransduction [77]. Future work should address the role of NuMA1 in the nucleus compartment, related to transcriptional activity within muscle cells.

### Nuclear mispositioning and myopathies

Numerous muscle diseases are characterized by alterations of myonuclei shape and myonuclear domain. In DMD, the absence of dystrophin leads to the most frequent form of muscular dystrophy, with repetitive degeneration/regeneration cycles that progressively trigger accumulation of damaged myofibers with aberrant myonuclei positioning [78]. Dystrophin is also described as a MAP and accordingly, in DMD animal models, alterations of MT network and myonuclei motion has been observed [31, 63, 79] and we confirmed this impairment in the present study (Fig. 1). Moreover, we demonstrated that nuclear NuMA1 contents are elevated in the *mdx* mouse model and in DMD patients (Fig. 6). Our experiments suggest that NuMA1 accumulation in myonuclei could be considered a marker of both myonuclei dynamic within myofibers and alterations in MT network integrity. Interestingly, we demonstrated that modulation of AMPK activity can directly affect nuclear NuMA1 accumulation and influence myonuclei dynamics, with beneficial effects on myonuclear spreading, when pharmacologically activated using a specific AMPK activator, such as the Benzimidazole derivative small-molecule 991 (Fig. 6). As AMPK activation has previously been shown to have beneficial effects on muscle function in dystrophic mice [54–56], our work highlights a new positive role for AMPK in muscle regeneration that goes beyond its previously described role in the regulation of inflammation [80–82]. Such actions should be further investigated through the study of NuMA1 muscle-specific activity and extended to other myopathies characterized by myonuclei mispositioning, such as CNMs and related diseases [5]. Future work should address how cytoplasmic NuMA1 activity is spatially regulated along muscle fibers and how it contributes to changes in MND in these myopathies. Our findings explain why such diverse phenotypes regarding myonuclei positioning can occur in these myopathies, maintaining aberrant myonuclear localization and, consequently, compromising myofiber integrity.

## Materials and methods

### Reagents

**Table:** 

siRNA	Sense oligonucleotide sequence	Anti-Sense oligonucleotide sequence
Scramble	Ambion, AM4641	
*Numa1*	GGGCAAGGUGAAGCAUUAA	UAAUGCUUCACCUUGCCC

shRNA	Clone Name	Target sequence
Scramble	SHC002 (MERCK)	Non-target sequence
*Numa1*	TRCN000007230 (Sigma)	GCCAGATGGATCGAAAGATTA

Plasmids	
EB1-GFP	From Edgar Gomes’ lab (institut myology, Paris, France)
RFP-Lamin-chromobody®	Chromoteck (lcg)
hNuMA1-WT-GFP	From Andreas Merdes’ lab (Center de Biologie Intégrative, Toulouse, France)
hNuMA1-S1853A-GFP	Directed mutagenesis performed in the lab
hNuMA1-S1853D-GFP	Directed mutagenesis performed in the lab
hNuMA1-NES	Directed mutagenesis performed in the lab

### Antibody used for cell culture immunostaining

**Table:** 

Antibody	Species and utility	Dilution factor	Manufacturer and reference
ɑ-Tubulin (microtubules)	Mouse – Primary	1/500	Sigma T6074
Myosin Heavy Chain (MHC)	Mouse – Primary	1/10	DSHB
Actin	Mouse – Primary	1/500	Millipore – Mab1501
NuMA1	Rabbit – Primary	1/500	Abcam – EP3976
GM130	Mouse - Primary	1/100	BD Biosciences 610823
PCM1	Rabbit – Primary	1/500	Sigma - HPA023370
Pericentrin	Mouse - Primary	1/100	BD Biosciences 611815
ɑ-Actinin	Mouse - Primary	1/500	Sigma – A7811
Anti-Mouse Alexa Fluor A488	Goat - Secondary	1/1000	Thermofisher scientific – A-11029
Anti-Rabbit Alexa Fluor A555	Goat - Secondary	1/1000	Thermofisher scientific – A-21428
DAPI (nuclei/myonuclei)	Dye	1/5000	Thermofisher scientific D1306

### Antibody used for isolated myofibers

**Table:** 

Antibody	Species and utility	Dilution factor	Manufacturer and reference
DAPI (myonuclei)	Dye	1/5000	Thermofisher Scientific D1306
NuMA1	Rabbit – Primary	1/500	Abcam – EP3976
Tyrosinated Tubulin (Microtubules)	Rat – Primary	1/500	Andrieux’lab
Pericentrin	Mouse - Primary	1/100	BD Biosciences 611815
GM130	Mouse - Primary	1/100	BD Biosciences 610823
Anti-Mouse Alexa Fluor A488	Goat - Secondary	1/500	Thermofisher Scientific – A-11029
Anti-Rabbit Alexa Fluor A555	Goat - Secondary	1/500	Thermofisher Scientific – A-21428

### Antibody used for muscle transversal cross-section

**Table:** 

Antibody	Species and utility	Dilution factor	Manufacturer and reference
DAPI (myonuclei)	Dye	1/5000	Thermofisher Scientific D1306
Lamininɑ2	Rat - Primary	1/1000	Santa Cruz – sc-59854
NuMA1	Rabbit – Primary	1/500	Abcam – EP3976
Anti-Rat Alexa Fluor A488	Goat - Secondary	1/500	Thermofisher Scientific – A-11006
Anti-Rabbit Alexa Fluor A555	Goat - Secondary	1/500	Thermofisher Scientific – A-21428

### Antibody used for western blot

**Table:** 

Antibody (WB)	Species and utility	Dilution factor	Manufacturer and reference
ɑ-Tubulin	Mouse – Primary	1/1000	Sigma T6074
Actin	Mouse – Primary	1/1000	Millipore – Mab1501
NuMA1	Rabbit – Primary	1/1000	Abcam – EP3976
HSP90	Mouse – Primary	1/1000	Enzo life SPA830
Histone H3	Rabbit – Primary	1/1000	Cell signaling – 9715S
Anti-Mouse HRP	Goat - Secondary	1/5000	Invitrogen 65-6520
Anti-Rabbit HRP	Goat - Secondary	1/5000	Invitrogen 65-6120

### Primers forward (F) and reverse (R) for qPCR

**Table:** 

Primers	Sequence
NuMA1 F	AAGTATTGACAGCCTGGATCTGA
NuMA1 R	GGTACGAGGCAGCTTGCT
GAPDH F	AACTTTGGCATTGTGGAAGG
GAPDH R	ACACATTGGGGGTAGGAACA
MHY4 F	CAAGTCATCGGTGTTTGTGG
MYH4 R	TGICGTACTIGGGAGGGTTC
Gusβ F	GAGGATTGCCAACGAAACCG
Gusβ R	GTGTCTGGGGACCACCTTTGA
RPL41 F	GCCATGAGAGCGAAGTGG
RPL41 R	CICCTGCAGGCGTCGTAG

### Primers for directed mutagenesis

**Table:** 

**NuMA1** **-** **S1853A**	Forward	CTAGAGACTGAGTAGCGGAGGTGGCTCGCAG
	Reverse	CTGCGAGCCACCTCCGCTACTCAGTCTCTAG
**NuMA1** **-** **S1853D**	Forward	AGCTAGAGACTGAGTATCGGAGGTGGCTCGCAGG
	Reverse	CCTGCGAGCCACCTCCGATACTCAGTCTCTAGCT

### Animals and animal experiments

Two-months-old male DMD^mdx4Cv^ mice (*mdx*) [83] with C57BL/6 background were used. As a control WT mice, we used C57BL/6 2-month-old mice. Three-month-old male *mdx* mice were treated with metformin (Sigma). Metformin was added to the drinking water for 3 weeks (200 mg/kg/day, i.e., 1 mg/ml replaced every day). The first 2 weeks of treatment was concomitant with fibrosis establishment in the fib-*mdx* model [57]. AMPKɑ1 WT or KO pups were used to performed primary culture experiment. Heterozygous animals were bred and AMPKɑ1 pups were genotyping, and myoblasts cells were plate dependent of the genotype of interest WT or KO. 2 months-old adult AMPKɑ1 WT or KO were used in uninjured condition.

For muscle injury experiment, 2-month-old male, Pax7-CreER^T2/+^; AMPKɑ1^fl/fl^ were used [51]. CreER^T2^ was activated by 2 mg/mouse tamoxifen (MP Biochemical – 02156738-CF) injection during 4 consecutive days, 1 week before muscle injury. To perform skeletal muscle injury, intramuscular cardiotoxin (Latoxan, L8102-1MG) injection was performed in *Tibialis Anterior* of mice. Mice were anesthetized with isoflurane inhalation and 50 µl of cardiotoxin was injected. 1x PBS as injected in control mice. Muscles were harvested 28 days post-injury.

All animal experimentations were approved by the French and European legislation. At the end of the procedure, mice were euthanized by an excess of isoflurane inhalation followed by cervical dislocation.

### Cell culture

#### Primary murine cells

Primary mouse myoblasts were purified from wild-type C57BL6 newborn mice as described before [30]. Hindlimb muscles from 4 to 7 days pups were extracted and digested with Collagenase I (Sigma, C9263-1G) and Dispase II (Roche, 04942078001). After 4 h of pre-plating to discard contaminant cells such as fibroblasts, myoblasts were cultured on 1% growth factor reduced Matrigel^®^/IMDM coated-dish (Corning, 356231; Ibidi µ-dish 35 mm high ibiTreat, 81256 or µ-Slide 4 Well ibiTreat, 80426) in growth media (IMDM (Gibco, 21980065), 20% fetal bovine serum (Gibco, A5256701), 1% chicken embryo extract (USBiological, C3999), 1% penicillin-streptomycin (Gibco, 15140-122), 0,5% gentamycin and 5% fibroblasts growth factor 2 (Milteny Biotec, 130-105-786)). Myoblasts were induced to differentiate in myotubes when they reached 80% of confluence for 2 or 3 days in differentiation media (IMDM (Gibco, 21980065), 2% horse serum (Gibco, 16050-22) and 1% penicillin-streptomycin (Gibco, 15140-122)). Myotubes were then covered by a layer of 50% growth factor reduced Matrigel^®^/IMDM (Corning, 356231) and maintain in culture up to 10 days in long differentiation culture media (IMDM (Gibco, 21980065), 2% horse serum (Gibco, 16050-22), 1% penicillin-streptomycin (Gibco, 15140-122) and 0,1% Agrin) until the formation of mature and contracting myofibres. Half of medium volume was changed every two days.

#### Primary human cell culture for immunofluorescence

Human primary myoblasts were isolated from uninjured semitendinosus muscles collected as surgical waste (protocol CCER no. PB_2016-01793 approved by the Swiss Regulatory Health Authorities and sanctioned by the “Commission Cantonale d’Ethique de la Recherche” of the Geneva Cantonal Authorities, Switzerland). Myoblasts were purified and cultured as described earlier [84]. Cells were seeded on a NanoSurface dish (ANFS-1, NanoSurface Biomedical, Inc.) coated with Matrigel (BD, diluted 1/100 in Ham’s F-10, ThermoFisher). After four days of differentiation, a second layer of Matrigel (BD, diluted 1/3 in differentiation medium) was added to cover the myotubes [85]. Half of the medium was replaced every other day.

#### Primary human cell culture for cell fractionation

Primary human myoblasts were plated on 1% Matrigel-coated dishes and cultured in a proliferative medium containing 40% skeletal muscle cell growth medium with supplemented mix (Promocell, C-23060), 30% DMEM high glucose and pyruvate (Gibco, 41966029), 20% decomplemented FBS (Gibco, 10270-106), 9% medium 100 GlutaMAX supplement (Gibco, 41150-020), 1% penicillin-streptomycin (Gibco, 15140-122), 0,005% Puromycin and 0,003% dexamethasone. Differentiation was induced by a switch into differentiation media (skeletal muscle cell growth medium, Promocell, C-23060). Differentiation medium was changed every other day.

#### C2C12 immortalized cells

Mouse myoblasts C2C12 cells were cultured in Dulbecco’s modified Eagle’s medium (DMEM (Gibco, 41966029) + 15% fetal bovine serum (Gibco, 10270-106) + 1% penicillin-streptomycin (Gibco, 15140-122)) and were plated on 1% matrigel-coated dishes for 1-2 days before induction of differentiation by switching into differentiation media (DMEM + 1% horse serum).

#### Cell transfection

Primary myoblast cells were transfected with either Lipofectamine 2000 (ThermoFisher Scientifics, 11668-019) for siRNA transfection at the final concentration of 10 nM, or with Lipofectamine 3000 (ThermoFisher Scientifics, 11668-019) for overexpression plasmids or shRNA transfection at the final concentration of 750 ng/µl, in accordance with manufacturer instructions, just before differentiation. Co-transfection experiments (i.e., plasmids and siRNA) were performed using Lipofectamine 2000 (ThermoFisher Scientifics, 11668-019) in accordance with manufacturer instructions.

#### AMPK pathway activation and inhibition

Three-day WT primary myotubes were treated with ADaM-site binding small molecules (991, Selleckchem – S8654), an allosteric AMPK activator. 10 µM of 991 was added in differentiation medium for 1 h at 37 °C and cells were fixed. Three-day *mdx* primary myotubes were treated either with DMSO, 10 µM of 991 or 1 µM of BAY3827 (AMPK inhibitor) for 1 h to measure nuclear NuMA1 content or for 24 h to see the effect on myonuclei positioning.

#### Purification of microtubule-associated proteins

We purified Microtubule-Associated Proteins (MAPs) from 3-day differentiated mouse primary myotubes or 10-day differentiated mouse primary myofibers as described before [86]. Myotubes or myofibers were detached using trypsin/EDTA 1X (Thermofisher Scientific 15400054). Cell pellet was diluted in an optimal volume of cold PEM [87] (0.5 M of PIPES pH 6.8, 2 M of MgCl_2_, 0.5 M EGTA). Cells were sonicated, kept 10 min on ice and lysates were homogenized using QIAshredder (Qiagen, 79656) before being clarified using centrifugation at 45,000 rpm with Bockman rotor TLA100.3, at 4 °C. To link MAPs and molecular motor to the MT network, clarified extract was put at 37 °C in a water bath and MTs were stabilized with Taxol (Molecular Probes, Cat. No. P-3456). 1 mM of GTP and 1.5 mM of AMP-PNP (adenylyl-imidophosphate) were added to the suspension for 90 min at 37 °C in a water bath. Solution of MTs and cell extracts were then put on 40% of glycerol cushion with 10 µm of Taxol diluted in PEM, centrifuged at 45,000 rpm for 20 min at 37 °C. For 3 times, MTs, MAPs and molecular motors were diluted in appropriate volume of warmed PEM and centrifuged at 45,000 rpm, 10 min at 37 °C. The final pellet was resuspended in water with Bolt reducing agent (Thermofisher Scientific, B0009) and Bolt LDS Buffer (Thermofisher Scientific, B0007). Spectrometry analysis was performed on a single mix of 3 experiments for each time point (3 days, myotubes, and 10 days, myofibers).

#### Mass spectrometry

Proteins were solubilized in Laemmli buffer and stacked in the top of a 4–12% NuPAGE gel (Invitrogen). After staining with Coomassie blue R-250 (Bio-Rad), they were digested in-gel using modified trypsin (Promega, sequencing grade) as previously described [88]. The resulting peptides were analyzed by online nanoliquid chromatography coupled to MS/MS (Ultimate 3000 and LTQ-Orbitrap Velos Pro, Thermo Scientific). For this purpose, the peptides were sampled on a precolumn (300 μm × 5 mm PepMap C18, Thermo Scientific) and separated in a 75 μm × 250 mm C18 column (PepMap, 3 μm, Thermo Scientific) using a 120-min acetonitrile gradient. The MS and MS/MS data were acquired using Xcalibur (Thermo Fisher Scientific).

Peptides and proteins were identified by Mascot (version 3.1.0, Matrix Science) through concomitant searches against the Uniprot database (*Mus musculus* taxonomy) and a homemade database of contaminant proteins classically found in proteomic analyses (human keratins, porcine trypsin, bovine albumin…). Trypsin/P was chosen as the enzyme and two missed cleavages were allowed. Precursor and fragment mass error tolerances were set at respectively at 10 ppm and 0.6 Da. Peptide modifications allowed during the search were: Carbamidomethyl (C, fixed), Acetyl (Protein N-term, variable) and Oxidation (M, variable). The Proline software [89] (version 2.3.3) was used for the compilation, grouping, and filtering of the results (conservation of rank 1 peptides, peptide length ≥6 amino acids, false discovery rate of peptide-spectrum-match identifications <1% [90], and minimum of one specific peptide per identified protein group). Proline was then used to perform a weighted spectral counting (WSC)-based comparison of the samples. Proteins were considered enriched in a sample compared to another if they were identified by at least 3 WSC and identified only in one sample or enriched at least 5 times in a sample compared to the other on the basis of WSC.

#### siRNA screening

As described before, primary mouse myoblasts were cultured on 1% growth factor reduced Matrigel^®^/IMDM coated 96 wells plate (UGAP, 2582249). Cells were transfected with Lipofectamine 2000 (ThermoFisher Scientifics, 11668-019). For each condition, 3 siRNAs are mixed at the final concentration of 10 nM in accordance with manufacturer instructions. Primary myoblasts were put in differentiation media for 3 days before fixation in 4% PFA (Thermoscientific 043368.9 M)/ 4% sucrose (Acros organics 177140010) in PBS for 20 min at 37 °C and immunofluorescence protocol followed.

### Immunofluorescence

#### On primary cells

Cells were fixed in 4% paraformaldehyde (Thermoscientific 043368.9 M)—4% sucrose (Acros organics 177140010)—0.1% TritonX-100 in PBS for 20 min at 37 °C followed by washes with PBS and membranes permeabilization with 0.5% Triton-X100 in PBS for 5 min at Room Temperature (RT). Following washes with PBS, cells were saturated with 1% BSA in PBS for 30 min at RT and incubated in primary antibodies overnight at 4 °C. Following washes with 0.05% Triton-X100 in PBS, cells were incubated in secondary antibodies or dyes for 1 h at RT followed by washes with 0.05% Triton-X100 in PBS and a last wash in PBS.

#### On isolated myofibers

Following dissection of the whole muscle from the mice, *Tibialis Anterior* muscles were fixed in 4% PFA (Thermoscientific 043368.9 M)/ 4% sucrose (Acros organics 177140010) in PBS for 2 h at RT. After washes, mono-myofibers were isolated from each muscle for each condition. Myofibers were permeabilized using 1% Triton-X100 in PBS for 15 min at 37 °C and saturated in 1% BSA in PBS for 30 min at RT. Myofibers were then incubated in primary antibodies at 4 °C for two nights. Following 0.05% Triton-X100 in PBS washes, myofibers were incubated in secondary antibodies or dyes overnight at 4 °C. Then, washes with 0.05% Triton-X100 in PBS and a last wash in PBS were carried. Myofibers were mounted on slides using Fluromount Aqueous mounting (Sigma, F4680) and kept at 4 °C. For some conditions, myofibers were incubated with pre-warmed homemade antigen retrieval buffer (10 mM Sodium Citrate (Sigma-Aldrich, C3434), 0.05% Tween-20 (Sigma-Aldrich, P1379), pH 6.0) for 10 min at 95 °C in steam cooker. Myofibers were then cooled to RT before three washes in PBS 1X. Myofibers were permeabilized in 0.5 M glycine, 0.5% Triton X-100, PBS 1X for 10 min at RT. Samples were then incubated in blocking solution containing 5% horse serum (Gibco, 16050122), 2% BSA (Euromedex, 04-100-812-E), 0.1% Triton X-100 PBS 1X for at least 45 min. Myofibers were subsequently processed as described above for immunostaining and mounting.

#### On isolated muscle transversal cross-section

*Tibialis Anterior*, muscles were collected, embedded in tragacanth gum, and quickly frozen in isopentane cooled in liquid nitrogen. Cross-sections (10 μm thick) were obtained from the middle portion of frozen muscles and processed for immunostaining. Muscle sections were fixed using 4% PFA (Thermoscientific 043368.9 M) and 4% sucrose (Acros organics 177140010) for 10 min at RT. After several PBS washes, permeabilization step was carried out using Triton X-100 0.1% for 10 min at RT. Sections were saturated in BSA 1% (Euromedex 04-100-812) for 1 h at RT. Primary antibodies were incubated overnight at 4 °C. The day after sections are incubated with secondary antibodies for 1 h at RT following by DAPI for 5 min. Sections were mounted on slides using Fluromount Aqueous mounting (Sigma, F4680) and kept at 4 °C.

### Images acquisition and analysis

#### Video microscopy, myonuclei, and EB1 tracking

##### Myonuclei tracking

Time-lapse images were acquired every 20 or 15 min, using Z1-AxioObserver (Zeiss) or Nikon AX confocal microscope (Nikon), 20× objective. Myonuclei were manually tracked using Metamorph (Zeiss) or Icy software, respectively and analyzed using SkyPad® plugin as described before [91].

##### EB1 tracking

Time-lapse images were acquired every 150 milliseconds using Ti Eclipse (Nikon) microscope 60X oil objective, Plan Apo, NA: 1.4, WD: 0.13, coverglass thickness: 0.17. EB1 comets trajectory were extracted using ImageJ®. To determine EB1 comets orientation, six continuous frames of a time-lapse movie of EB1-GFP were overlapped, the first three are color-coded in green and the last three are color-coded in red. EB1 comets velocity was measured using Particle Tracker in ImageJ^®^[92].

#### High-resolution microscopy (SIM)

Following dissection of the whole muscle from the mice, *Tibialis Anterior* muscles were fixed in 4% PFA (Thermoscientific 043368.9 M)/4% sucrose (Acros organics 177140010) in PBS for 2 h at RT. After washes, mono-myofibers were isolated from each muscle for each condition. Heat-induced epitope retrieval was done with steaming method in Antigen Unmasking Solution, Tris-Based (H-3301), 20 min. After PBS washes, a blocking step was performed with saturation solution (1X PBS, 0.05% Triton-X100, 3% Bovine Serum Albumin, 5% Normal Goat Serum) for 45 min at RT. Myofibers were then incubated with primary antibodies diluted in saturation solution at 4 °C for 4 nights. Following 0.05% Triton-X100 in PBS washes, myofibers were incubated with secondary antibodies overnight at 4 °C. Then, washes with 0.05% Triton-X100 in PBS and a last wash in PBS were carried. Myofibers were deposited on 25 mm coverslip 1.5H (Marienfeld, ref:0117650) coated with 5 mg/mL gelatin from bovine skin and 1 mM chromium (III) potassium sulfate to ensure fibers were positioned near the objective to minimize surface irregularities that could compromise resolution. Coverslips were then mounted with Prolong Glass (Fisher ref 15810993).

3D Lattice-SIM images were acquired on a Zeiss Elyra 7 AxioObserver microscope equipped with a Plan-Apochromat 63×/1.46 oil objective and a Photonlines pco.edge 4.2 sCMOS camera and controlled by the software Zen 3.0 SR Black. For fluorescence signal detection, 405 nm (100 mW), 488 nm (100 mW), 561 nm (100 mW) and 640 nm (500 mW) laser lines were used respectively with the filters (nm) BP 420-480_LP 650/ BP 420-480_495-525_LP 650 / BP 495-550_570-620/ BP 420-480_LP 650. 3D Lattice-SIM acquisitions were performed using 13 pattern translations. Z-stacks were acquired using 100 nm intervals. The raw image obtained has a pixel size of 62.61 nm and post processing image has a pixel size of 31.3 nm. Exposure time and laser power were balanced for each fluorescence channel individually to minimize bleaching and exposure time. 3D Lattice-SIM processing was performed using the SIM processing tool from the Zen 3.0 SR Black software.

#### Confocal microscopy

Isolated myofibers, muscle transversal cross-section, and primary cells images were acquired using Z1-AxioObserver (Zeiss) or LSM 880 confocal microscope (Zeiss) or Nickon AX microscope (Nikon) with 60X oil objective.

#### Image analysis

##### Myonuclei spreading analysis in myotubes

Quantifications in immature myotubes were assessed using an analysis tool developed in our team. An image analysis performed in ImageJ® software is combined with a statistical analysis in RStudio® software. This provides quantifications of parameters, ranked by myonuclei content per myotubes, regarding phenotype of myotubes (area, length) and their respective myonuclei positioning compared to centroid of myotubes (DMcM). MSG diagrams were obtained through the normalization of lengths of all analyzed myotubes (independently to their myonuclei content) to 100%. White lines represent myonuclei density curves assessing the statistical frequency for myonuclei positioning along myotubes. Each color group reflects statistical estimation of myonuclei clustering along myotubes.

##### Myonuclei shape, MND organization, and NuMA1 intensity

Myonuclei shape was analyzed using Fiji® software. Myonuclei were segmented based on nuclei staining (DAPI) and myonuclei circularity, area, and nuclear NuMA1 intensity parameters were extracted. MND organization was analyzed using Fiji® software by measuring the distance between the closest myonuclei.

##### Myonuclei localization in primary myotubes/myofibers

We considered as a 3-day primary myotubes, each structure MHC positive that contain at least 6 myonuclei. In each individual myotube, we considered aligned myonuclei when 70% of myonuclei are well aligned in the center of myotube length. The percentage of myotubes with aligned or aggregated myonuclei was calculated based on the total number of myotubes in the picture.

We considered 10-day primary myofibers, each structure that contain striated actin staining. The percentage of events with isolated and centralized myonuclei, aggregated or peripherized myonuclei, was calculated.

##### Fusion index

The fusion index was determined by the ratio of the number of myonuclei in MHC-positive structures (i.e., 3-day primary myotubes) on the total number of nuclei in the picture.

##### MT architecture organization

MT architecture organization was analyzed using TeDT software as previously described [93].

#### Protein sample preparation from cell culture

Cells were harvested using trypsin (Gibco, 15400054) for 5 min at 37 °C and centrifuged at 1500 rpm for 5 min at 4 °C. Cell pellets were diluted and incubated in the optimal volume of RIPA lysis buffer (homemade), containing phosphatase inhibitors (Sigma, P5726) and protease inhibitors (Sigma, P8340), for 10 min at 4 °C. Following a sonication step at 4 °C, protein samples were collected for further use. The concentration of proteins was determined using BCA protein assay kit (Thermo Fisher Scientifics, 23225) as described by the manufacturer.

#### Subcellular fractionation assays from cell culture

Cells were harvested, using trypsin for 5 min at 37 °C and centrifuged at 1500 rpm for 5 min at 4 °C. Cell pellet was diluted on E1 lysing buffer (NaCl 140 mM, glycerol 10%, NP40 0.5%, Triton X-100 0.25%, DTT 1 mM, EDTA 1 mM, Hepes-KOH pH7.5 500 mM, phosphatase inhibitors (Sigma, P5726) and protease inhibitors (Sigma, P8340)). After a centrifugation step of 10 min at 5000 × *g*, supernatant containing the cytoplasmic fraction was collected in a new tube. Pellet was diluted on the same volume of E1 buffer as mentioned before and centrifuged for 10 min at 5000 × *g* at 4 °C. This step was repeated with an additional step of 10 min on ice before the centrifugation and removing supernatant. Pellet was diluted on SDC buffer (NaCl 600 mM, NP40 1%, SDS 0.1%, Tris-HCl pH7.5 50 mM, sodium deoxycholate 0.5%, phosphatase inhibitors (Sigma, P5726) and protease inhibitors (Sigma, P8340) following by a sonication step at 4 °C, samples were incubated for 30 min on ice, corresponding to the nuclear fraction. All fractions were centrifuged at 10,000 × *g* for 10 min at 4 °C and protein samples were collected for further use.

#### Western blot

Samples were loaded onto 6% acrylamide gels and migrated at 100 V for 10 min followed by 90 min at 130 V. Wet blotting system (BioRad) was used to transfer proteins to PVDF membranes (Millipore, Immobilon-P, IPVH00010). Membranes were then saturated in 5% BSA in 0.1% Tween20 – 1X TBS for 1 h at RT and were incubated in primary antibodies in 5% BSA in TBS overnight at 4°C. Following washes by 0.1% Tween20 – 1X TBS, membranes were incubated in HRP-conjugated secondary antibodies in 1% BSA in TBST for 1 h at RT. Following washes in 0.1% Tween2—1X TBS, detections of target proteins were carried out using clarity Western ECL substrate (BioRad, 1705060) and ChemiDoc imaging system (BioRad).

#### RNA extraction from cell culture

After addition of Trizol (Sigma, T9424), samples were incubated in chloroform for 5 min at RT, centrifuged for 15 min at 12,000 × *g* at 4 °C and incubated in tubes containing isopropanol for 10 min at RT. Samples were then centrifuged for 15 min at 12,000 × *g* at 4 °C, washed two times with 70% ethanol and RNA pellets were diluted into ultra-pure RNase-free water (Invitrogen, 10977-035). RNA concentration was analyzed using Nanodrop® (ThermoFisher Scientifics).

#### RT-q-PCR

Goscript Reverse Transcriptase System (Promega, A5001) was used as described by the manufacturer to produce the cDNAs. Fast Start Universal SYBR Green Master (Rox) (Roche, 04913914001) and CFX Connect™ Real-Time PCR Detection System (BioRad) were used to carry out quantitative PCR using the primers described in table below. Normalization was performed using housekeeping genes.

#### Mice electroporation

Eight-week-old male C57BL/6 WT mice were anesthetized with intraperitoneal injection of ketamine (100 mg/kg)—xylazine (10 mg/kg). Hind legs were shaved and 20 µg of plasmid DNA diluted into 0.9% NaCl was injected in both contralateral *Tibialis Anterior* muscles. Injected muscles received a short massage, and a thin layer of ultrasound gel was applied. Electrodes were positioned on both side of muscles and an electrical field was applied characterized by 8 pulses of 20 ms spaced 500 ms apart, at 200 V/cm of muscle thickness (i.e., 80 V). Two weeks post-electroporation, mice were sacrificed, and muscles were harvested. The whole experiment procedure is in accordance with the guidelines of the local animal ethics committee of the University Claude Bernard – Lyon 1 and in accordance with French and European legislation on animal experimentation and approved by the ethics committee CECCAPP and the French Ministry of Research.

#### Mutagenesis

Directed mutagenesis was performed to introduce a punctual mutation in NuMA1-WT plasmid. It was performed following manufacturer's instructions with QuickChange II XL site-directed Mutagenesis Kit (Agilent, 200521).

#### Human sample

Human biological samples and associated data were obtained from Tissu-Tumorothèque Est Biobank (CRB-HCL Hospices Civils de Lyon BB-0033-00046), authorized by the French Ministry of Research. (Collection Number CRB HCL: DC2019-3464). Written informed consent was obtained from all the patients of this study.

## Supplementary information

Supplementary Figure legends

Supplementary Table 1

Supplementary Table 2

Supplementary figure 1: Extended duration of mdx myonuclei movement and identification of MT-associated proteome involved in myonuclei positioning.

Supplementary figure 2: NuMA1 controls myonuclei positioning and myoblast fusion

Supplementary figure 3: Increased myonuclei pause duration and altered shape upon Numa1 down-regulation or overexpression of NuMA1-NES construct

Supplementary figure 4: NuMA1 in MT polymerization, nuclear MTOC organization and myonuclei localization in vivo

Supplementary figure 5: Nuclear/cytoplasmic NuMA1 content drives myonuclei dynamics

Supplementary figure 6: NuMA1–nMTOC proteins in mdx myofibers

Western Blot File

## Data Availability

All data generated or analyzed during this study are included in this published article and its supplementary information files and are available from the corresponding author on reasonable request.
