# Supplementary material for: NuMA1 controls myonuclear motility in striated skeletal muscle through AMPK activity and is impaired in Duchenne muscular dystrophy
Source: Cell Death Dis. 2026 Jun 17;17(1):758. doi: 10.1038/s41419-026-08987-5 (PMC13518885; doi:10.1038/s41419-026-08987-5)

Figure 3 E-F

NuMA1

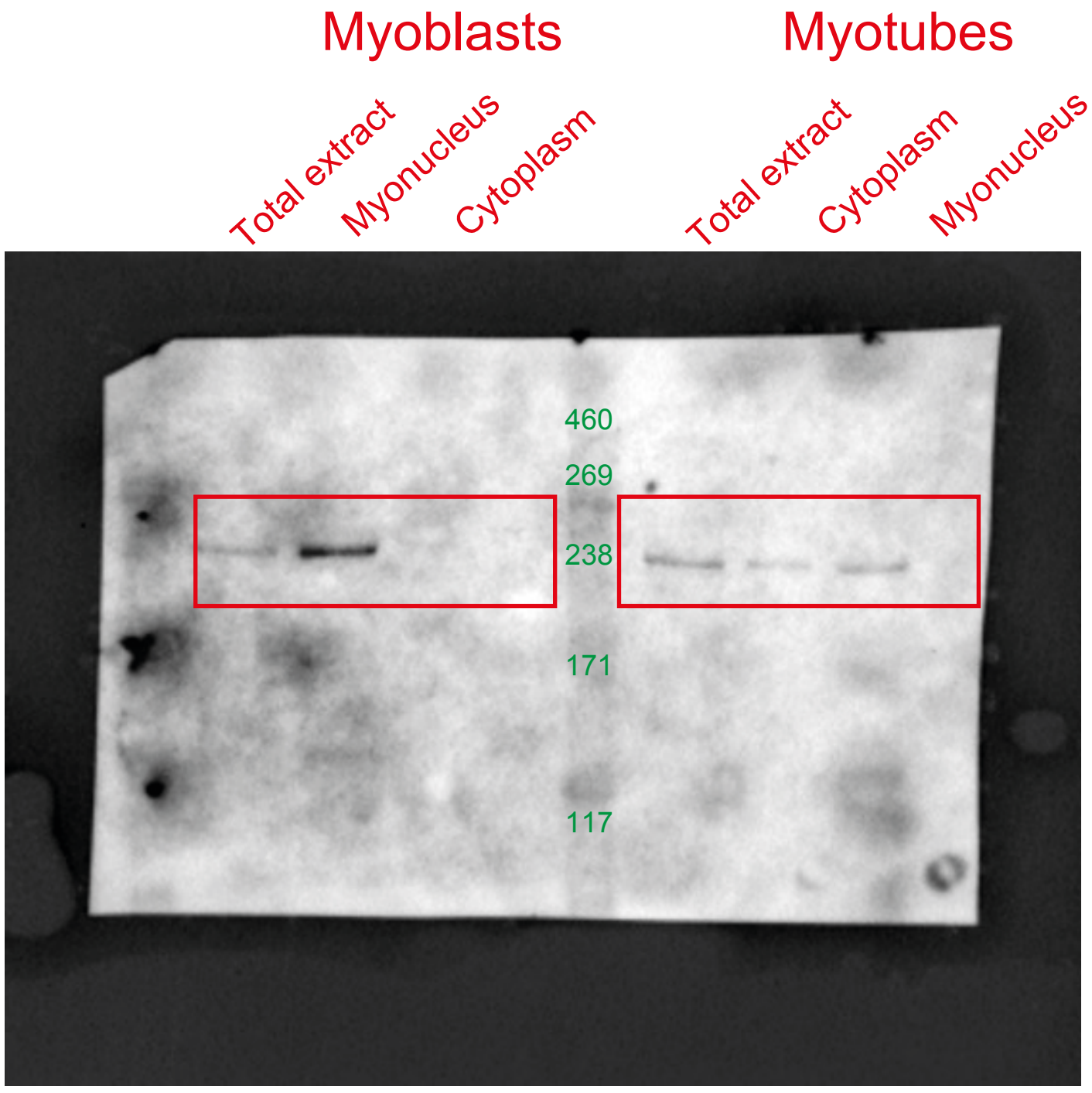

Histone H3

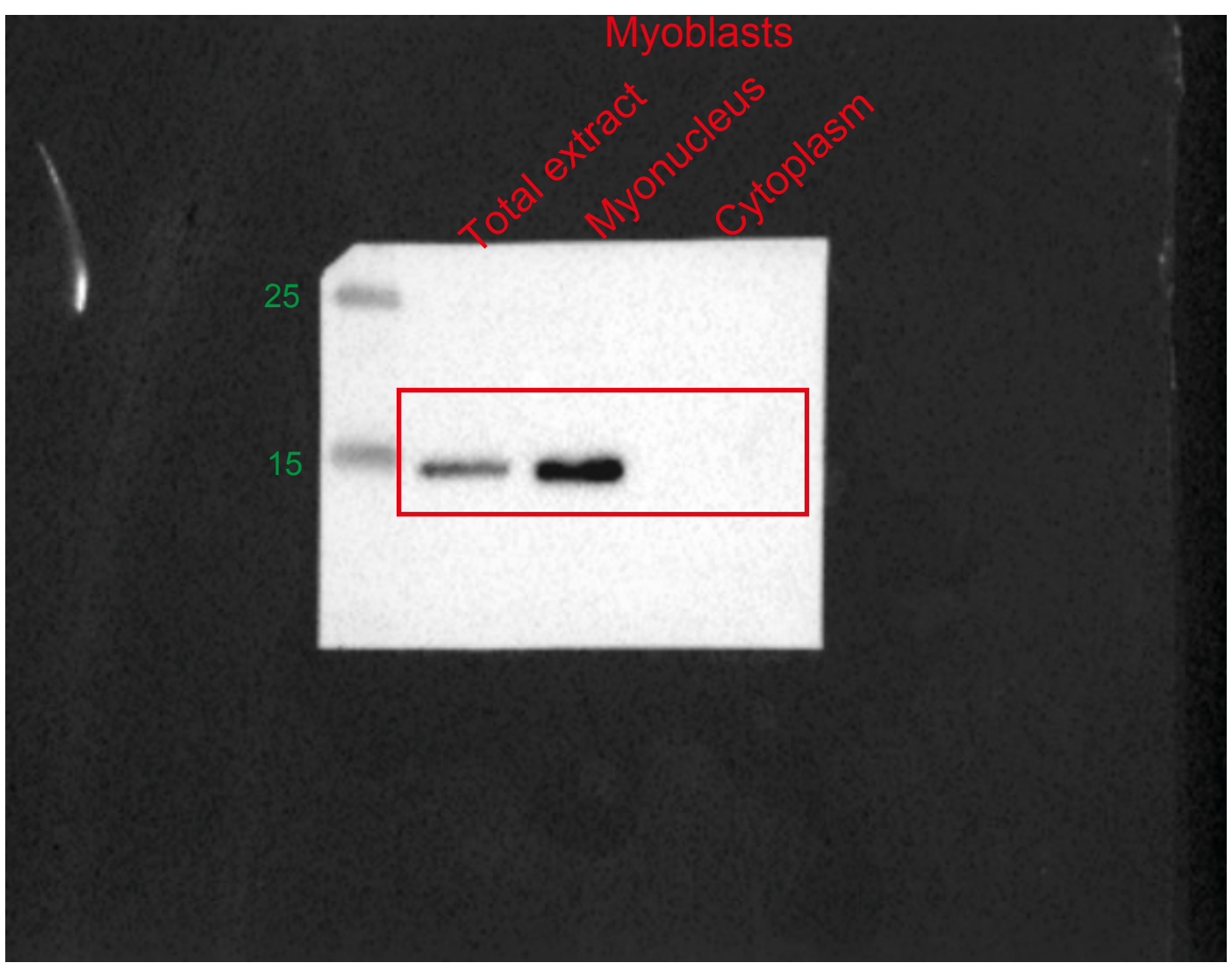

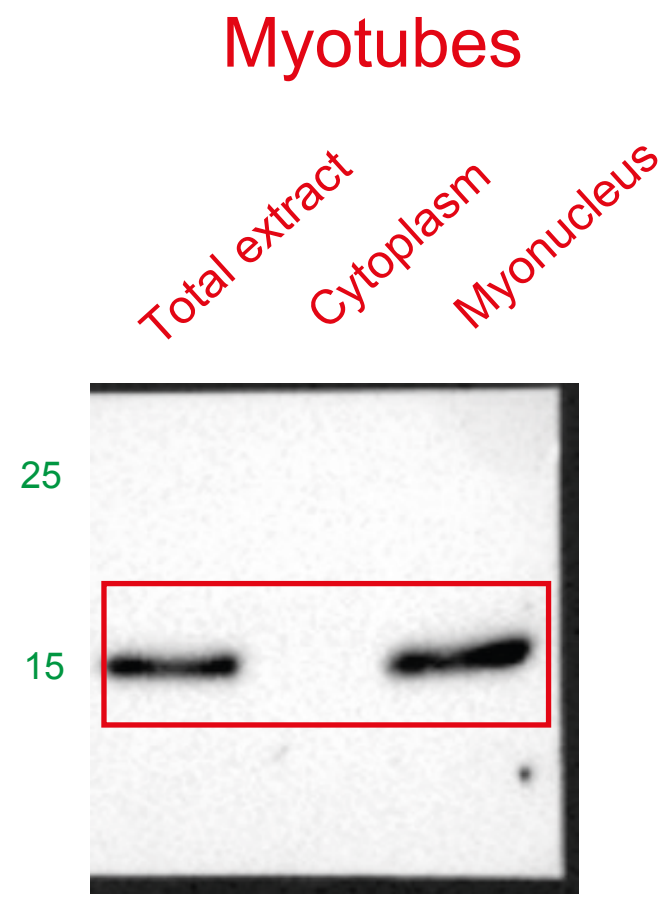

Histone H3

Tubulin

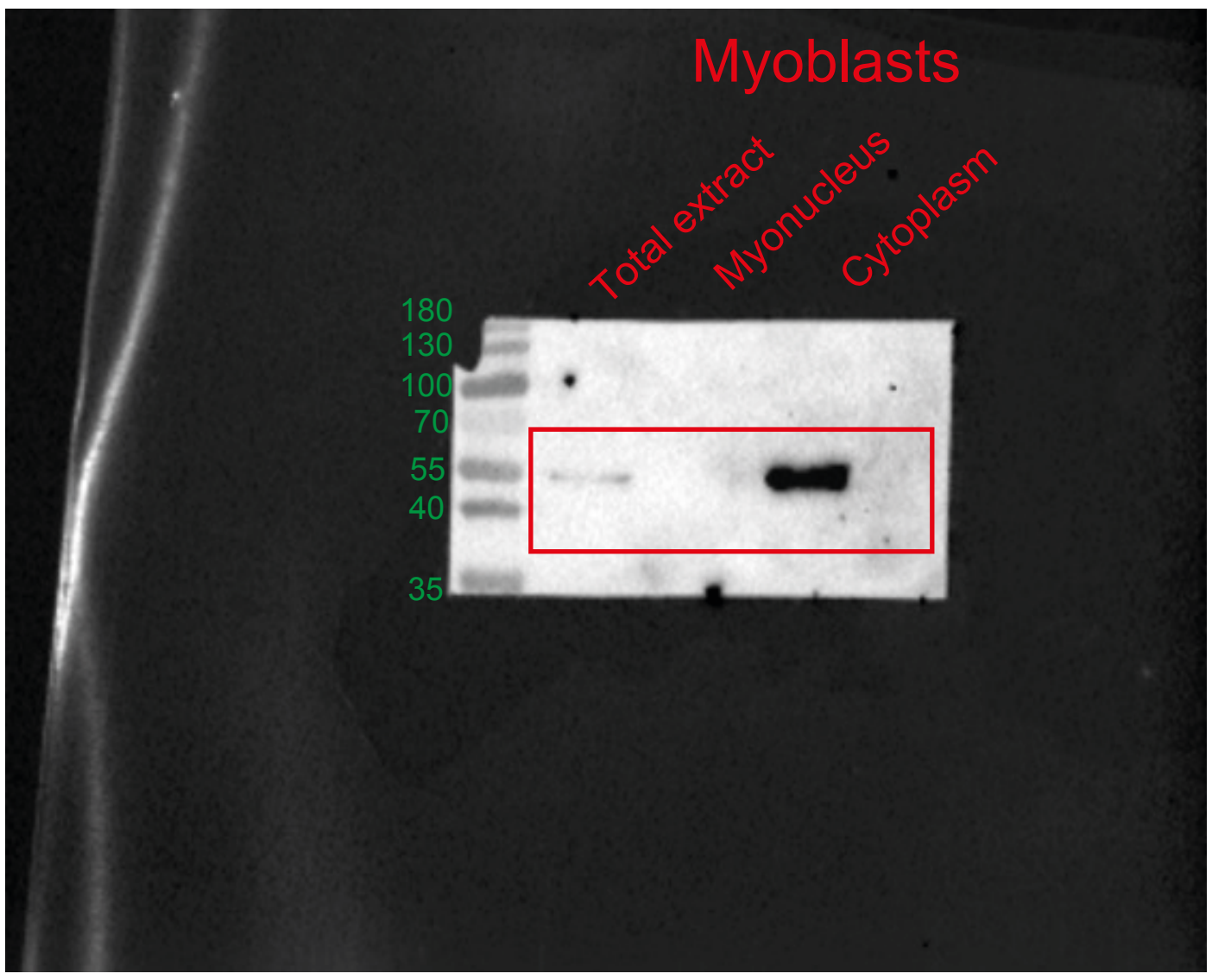

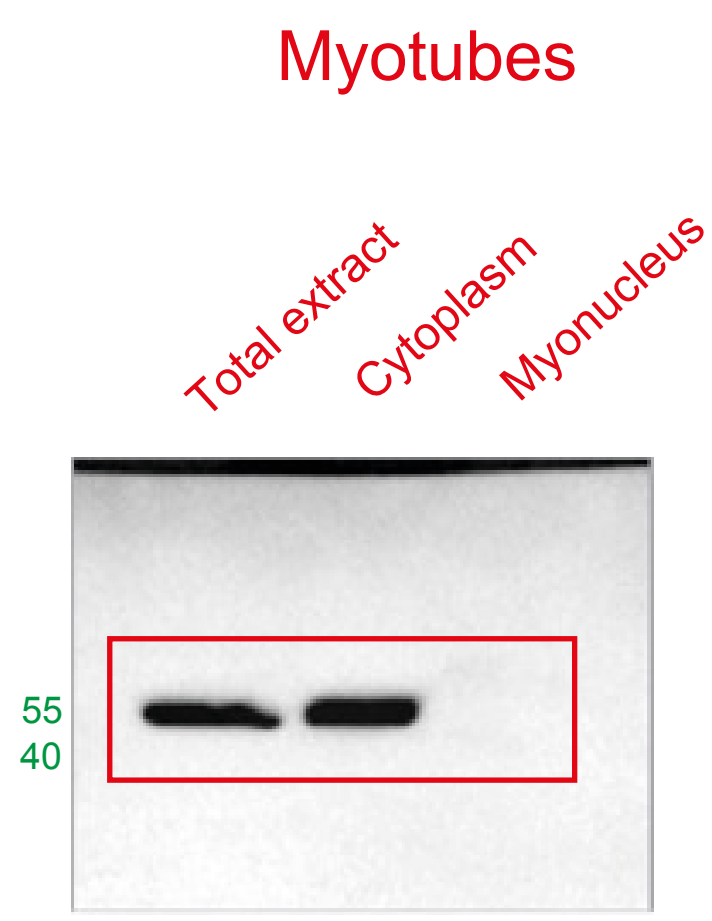

Tubulin

Supplementary Figure 2a

NuMA1

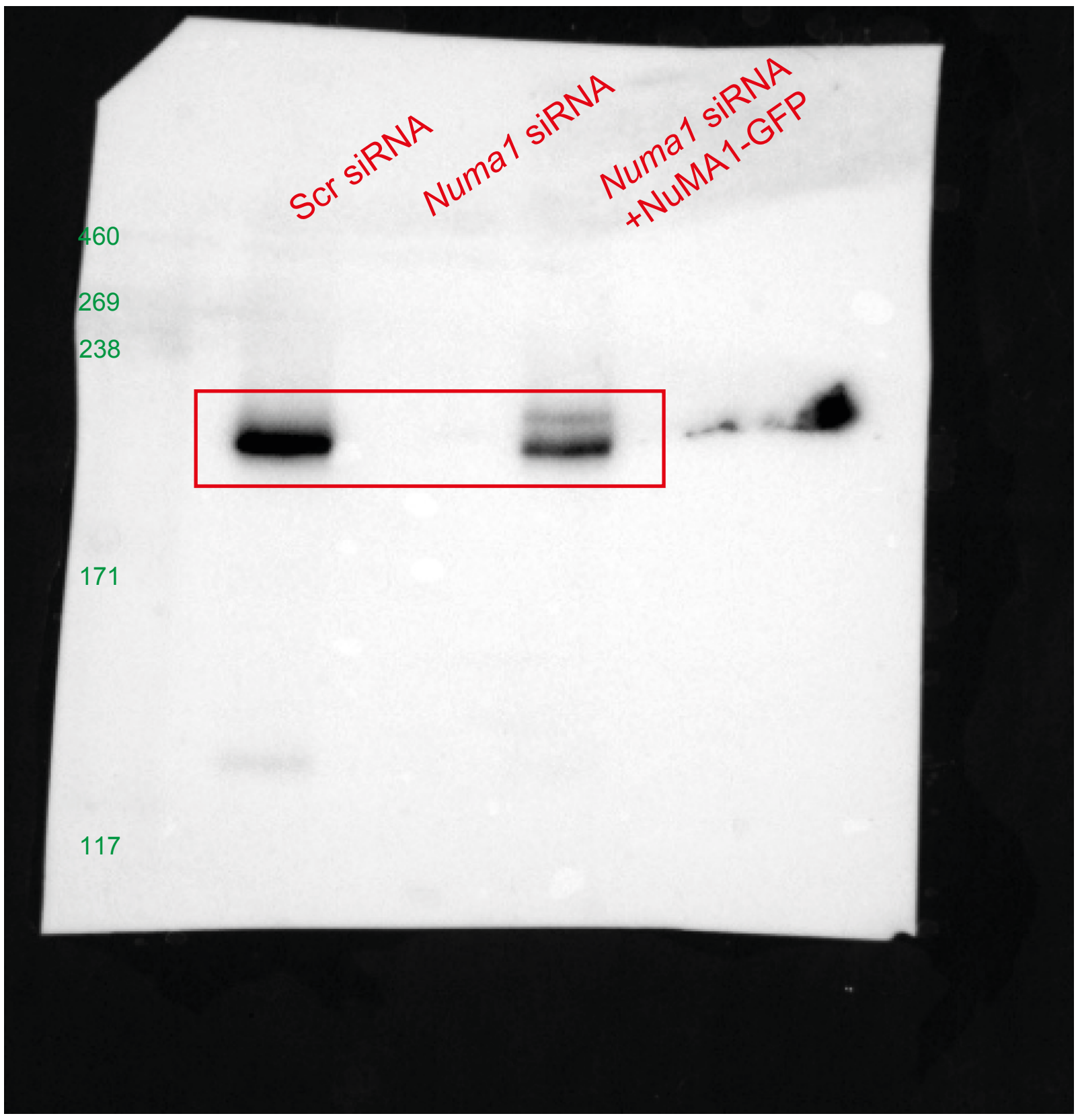

Actin

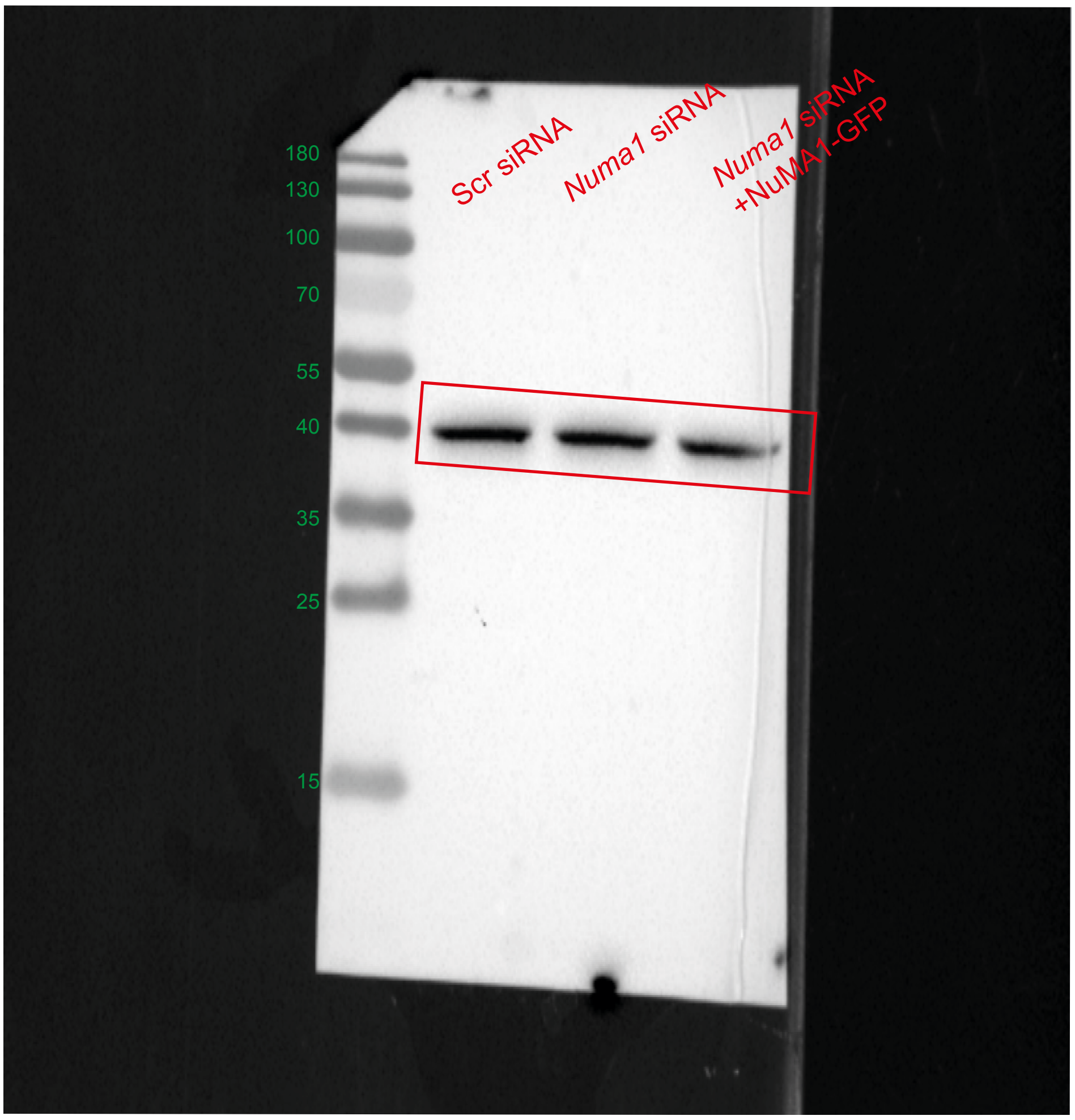

Supplementary Figure 3g

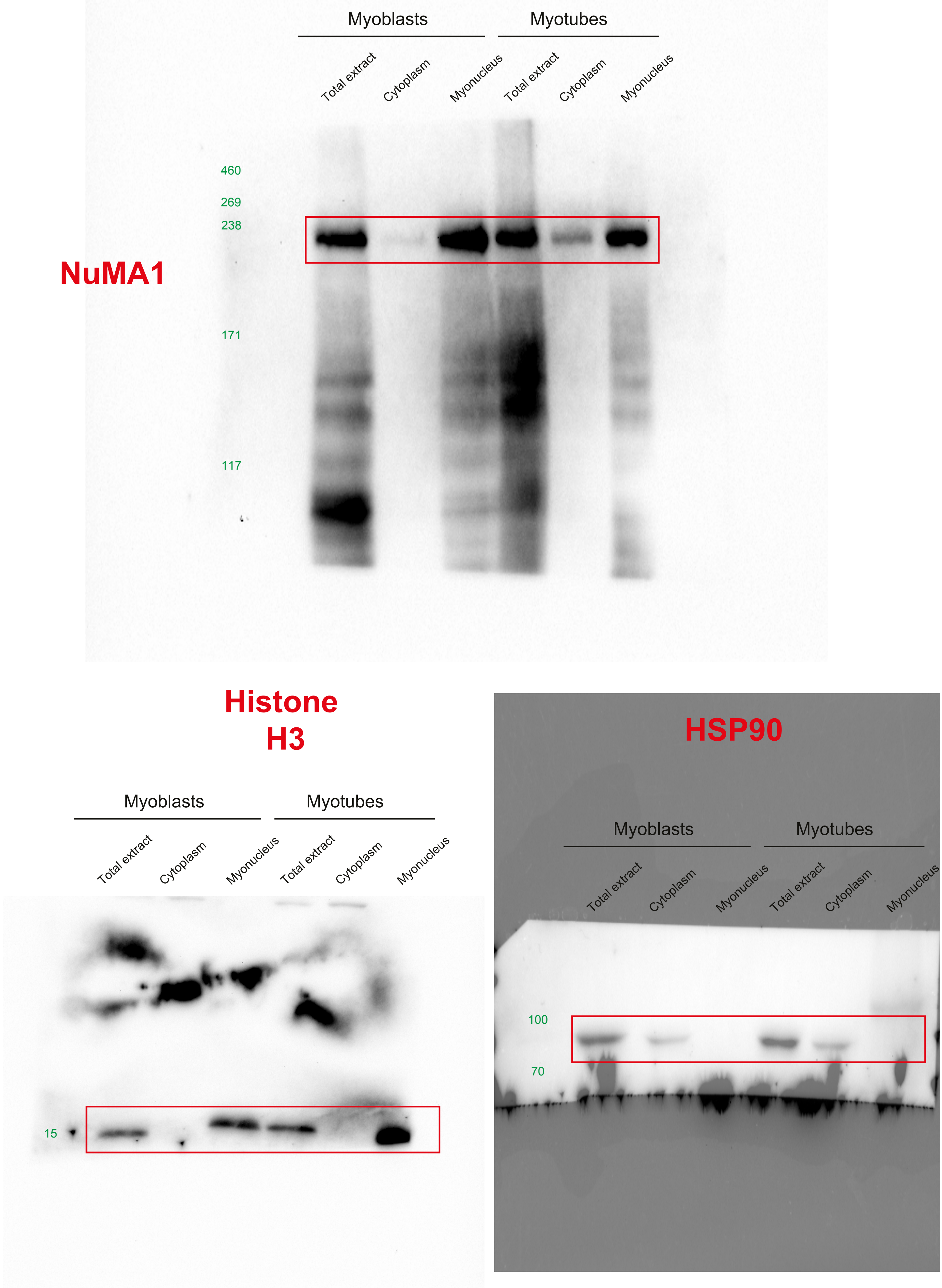

Supplement: Supplementary file 1 [file 41419_2026_8987_MOESM10_ESM.pdf]
