# Supplementary material for: NuMA1 controls myonuclear motility in striated skeletal muscle through AMPK activity and is impaired in Duchenne muscular dystrophy
Source: Cell Death Dis. 2026 Jun 17;17(1):758. doi: 10.1038/s41419-026-08987-5 (PMC13518885; doi:10.1038/s41419-026-08987-5)
Supplement: Supplementary file 2 [file 41419_2026_8987_MOESM1_ESM.docx]

**Supplementary figure 1:** **Extended duration of *mdx* myonuclei movement and identification of MT-associated proteome involved in myonuclei positioning.**

**a/b.** Quantification of persistence of myonuclei displacement (a.u.) (a) and total pausing time (min) (b) in 5-day WT or *mdx* primary myofibers *in vitro* (n=3 independent primary cultures, 300 WT and 239 *mdx* myonuclei tracked). **c.** Schematic of the biochemical procedure for purification and identification of proteins involved in myonuclei positioning in 3-day primary myotubes and 10-day primary myofibers *in vitro*. **d.** Distribution of partners identified by mass spectrometry; specific to 3-day myotubes, 10-day myofibers, or shared between both. **e.** Quantification of myonuclei spreading index of identified partners in 3-day myotubes, 10-day myofibers, or shared conditions *in vitro*. Graphical boxes showed the total data population represented by the higher and the lower point of the population. The median is represented. Unpaired T-Test were performed between groups. Each p-value is mentioned in the figure legend of each graph, ns (not significant) *p<0.05, **p<0.01 and ***p<0,001.

**Supplementary figure 2:** **NuMA1 controls myonuclei positioning and myoblast fusion**

**a.** Western-blot showing *Numa1* down-regulation in 3-day primary myotubes after scramble or *Numa1* siRNA transfection and rescue transfection with hNuMA1-GFP plasmid *in vitro*. **b.** Relative *Numa1* mRNA expression in 3-day primary myotubes after scramble or *Numa1* siRNA transfection. **c.** Quantification of myotubes length (µm) in 3-day primary myotubes after scramble or *Numa1* siRNA transfection, classified by myonuclei number per myotube (n=6 independent primary cultures, 618 scramble and 687 *Numa1* siRNA myotubes analyzed). **d.** Representative images of 3-day primary myotubes after scramble or *Numa1* siRNA transfection, stained for Myosin Heavy Chain (MHC, green) and nuclei (red) (Scale bar: 50 µm). **e.** Quantification of the percentage of 3-day primary myotubes with aggregated myonuclei after scramble or *Numa1* siRNA transfection (n=3 independent primary cultures). **f.** Representative images of 3-day primary myotubes after scramble, *Numa1* siRNA or rescue condition (*Numa1* siRNA + hNuMA1-GFP), stained for MHC (red) and nuclei or hNuMA1-GFP (green) (Scale bar: 50 µm). **g/h.** Quantification of percentage of 3-day primary myotubes with aligned myonuclei (g) and fusion index (h) after scramble, *Numa1* siRNA, or rescue transfection (n=3 independent primary cultures). Graphical boxes showed the total data population represented by the higher and the lower point of the population. The median is represented. Unpaired T-Test were performed between groups. Each p-value is mentioned in the figure legend of each graph, ns (not significant) *p<0.05, **p<0.01 and ***p<0,001.

**Supplementary figure 3: Increased myonuclei pause duration and altered shape upon *Numa1* down-regulation or overexpression of NuMA1-NES construct**

**a**. Quantification of total myonuclei pausing time (min) in 5-day primary myofibers after scramble or *Numa1* siRNA transfection (n=2 independent primary cultures, 214 scramble and 196 *Numa1* siRNa myonuclei tracked). **b.** Representative immunofluorescence images of 3-day primary myotubes after scramble or *Numa1* siRNA transfection, stained for myonuclei (gray) (Scale bar: 15 µm). **c-d.** Relative expression of *Numa1* (c) and *Myosin Heavy Chain 4* (d) mRNA during C2C12 differentiation (n=3 independent cultures). **e.** *Numa1* and *Myosin Heavy Chain 4* mRNA relative expression in 3-day primary myotubes and 10-day primary myofibers (n=3 independent primary cultures). **f.** Western-blot of 5-day human primary myotubes cell fractionation, probed for NuMA1; HSP90 and histone H3 used as cytoplasmic and nuclear loading controls, respectively. **g.** Representative immunofluorescence images of 5-day primary myofibers transfected with constructs expressing either WT-NuMA1-GFP with NLS sequence (NuMA1-NLS) or WT-NuMA1-GFP with NES sequence (NuMA1-NES), stained for GFP (gray/green), PCM1 (gray/blue) and Tubulin (red) (Scale bar: 10 µm). **h-j** Quantification of the median myonuclei speed (µm/min) (h), myonuclei movement (% of time) (i) and myonuclei distance travelled by each myonuclei for 12 h (j) of myonuclei expressing WT-NuMA1, either with a high (WTH), or a low (WTL) level of WT-NuMA1, and myonuclei expressing WT-NuMA1 in which the NLS is replaced by an NES sequence (WT-NES). (n=3 independent primary cultures, 95 myonuclei for WTH, 97 for WLT and 151 for WT-NES were analyzed). Graphical boxes show the total data population represented by the higher and the lower point of the population. The median is represented. Unpaired T-Test was performed between groups. Each p-value is mentioned in the figure legend of each graph, ns (not significant), *p<0.05, **p<0.01 and ***p<0,001.

**Supplementary figure 4: NuMA1 in MT polymerization, nuclear MTOC organization and myonuclei localization *in vivo.***

**a.** Representative timelapse images of 3-day primary myotubes co-transfected with EB1-GFP and either scramble or *Numa1* siRNA *in vitro* (Scale bar: 9 µm). **b.** Quantification of EB1-GFP comet velocity (µm/min) in 3-day primary myotubes with scramble or *Numa1* siRNA transfection (n=3 independent primary cultures, 1183 scramble and 2154 in *Numa1* siRNA comets tracked). **c/d**. Representative images of 3-day primary myotubes after scramble (c) or *Numa1* (d) siRNA transfection, stained for myonuclei (red) and GM130 (green) (Scale bar: 10µm)**. e.** Quantification of the percentage of myonuclei with partial or complete GM130 relocalization around myonuclei in primary myocytes or 3-day primary myotubes after scramble or *Numa1* siRNA transfection (n=3 independent primary cultures). **f.** Scheme of *Tibialis Anterior* muscle shRNA electroporation protocol and picture of representative’s electroporated shRNA-GFP (green) muscle fibers. **g.** Representative immunofluorescence images of transverse cross-sections of *Tibialis Anterior* muscles electroporated with scramble or shRNA targeting *Numa1* and stained for GFP (shRNA, green), laminin (red) and myonuclei (blue) Scale bars: 50µm. White arrows indicate myonuclei. **h.** Quantification of mis-oriented myonuclei in electroporated *Tibialis Anterior* muscle fibers with scramble or shRNA targeting *Numa1.* **i.** Distribution of cross-sectional area of *Tibialis Anterior* muscle fibers electroporated with scramble or shRNA targeting *Numa1.* Graphical boxes show the total data population represented by the higher and the lower point of the population. The median is represented. Unpaired t-test were performed between groups. Each p-value is mentioned in the figure legend of each graph, ns (not significant), *p<0.05, **p<0.01 and ***p<0,001.

**Supplementary figure 5: Nuclear/cytoplasmic NuMA1 content drives myonuclei dynamics**

**a.** Schematic of NuMA1 protein domains highlighting phosphorylation sites targeted by kinases. **b.** Quantification of fusion percentage in 3-day *Ampkɑ1* WT or KO primary myotubes (n=3 independent primary cultures, 15 mosaic images analyzed). **c/d.** Quantification of myonuclei speed (µm/min) (c) and total pausing time (min) in 5-day *Ampkɑ1* WT or KO primary myofibers (n=3 independent WT primary cultures with 536 analyzed myonuclei and n=4 independent KO primary cultures with 505 myonuclei tracked). **e.** Representative images of 3-day primary myotubes after hNuMA1-WT transfection, stained for plasmid-GFP (green) and myonuclei (red) (Scale bar: 10 µm) **f.** Quantification of myonuclei total pausing time (min) in 3-day primary myotubes in control or transfected with NuMA1-WT, NuMA1-S1853A, or NuMA1 S1853D (n=2 independent primary cultures for control, 215 myonuclei tracked ; n=3 independent primary cultures for mutants, 149 WT, 121 S1853A, and 109 S1853D myonuclei tracked). **g-h.** Quantification of cytoplasmic NuMA1 intensity (a.u.) in myofibers from *Soleus* muscles transversally cross-sectioned (g) or from mdx myotubes treated with DMSO, 991 or BAY3827 (h). Graphical boxes show the total data population represented by the higher and the lower point of the population. The median is represented. Unpaired t-test were performed between groups. Each p-value is mentioned in the figure legend of each graph, ns (not significant), *p<0.05, **p<0.01 and ***p<0,001.

**Supplementary figure 6: NuMA1–nMTOC proteins in *mdx* myofibers**

**a-b.** Representative immunofluorescence images of isolated *Tibialis Anterior* myofibers from 2-month-old WT or *mdx* mice, stained for Pericentrin (green, a) and GM130 (Golgi apparatus) (green, b). Blue arrows indicate polarized Pericentrin distribution in wt myofibers. (Scale bar: 10 µm).

**Supplementary Table 1: Mass spectrometry-based proteomic comparison of microtubule-associated proteins in primary myoblasts differentiated for 3 and 11 days**

Tab 1 contains accession numbers of protein name, gene name, molecular weight (Da), number of peptides identified and the % of coverage for each proteins identified. Details for each protein’s identification is mentioned according to either 3 or 11 days of differentiation as well as the ration between 11 and 3 days of differentiation. Tab 2 described the condition to classified proteins as enriched in 11 days or enriched in 3 days differentiated primary myoblasts and the color code for selected proteins screened in Supplementary Table 2. These data were used to build the pie chart in Supplementary Figure 1d.

**Supplementary Table 2: SiRNA screening of myonuclei spreading of specific proteins from the mass spectrometry-based proteomic comparison of microtubule-associated proteins**

Tab contains proteins names, number of myotubes counted in each condition, spreading coefficient and its percentage of variation compare to control condition. These data were used to build the histogram in Supplementary Figure 1e.
